# Characterization of a novel *Plasmodium falciparum* merozoite surface antigen and potential vaccine target

**DOI:** 10.3389/fimmu.2023.1156806

**Published:** 2023-04-14

**Authors:** Karamoko Niaré, Timothy Chege, Micha Rosenkranz, Kennedy Mwai, Zoe Saßmannshausen, Dennis Odera, Lydia Nyamako, James Tuju, Tiono Alfred, John N. Waitumbi, Bernhards Ogutu, Sodiomon B. Sirima, Gordon Awandare, Bourema Kouriba, Julian C. Rayner, Faith H. A. Osier

**Affiliations:** ^1^ Department of Biochemistry, Cell and Molecular Biology, West African Centre for Cell Biology of Infectious Pathogens, University of Ghana, Accra, Ghana; ^2^ Kenya Medical Research Institute (KEMRI)-Wellcome Trust Research Programme, Centre for Geographic Medicine Research—Coast, Kilifi, Kenya; ^3^ Malaria Research and Training Centre (MRTC), Department of Epidemiology of Parasitic Diseases, Faculty of Pharmacy, University of Sciences, Techniques and Technologies of Bamako, Bamako, Mali; ^4^ Department of Pathology and Laboratory Medicine, Brown University, Providence, RI, United States; ^5^ Centre for Infectious Diseases, Parasitology, Heidelberg University Hospital, Heidelberg, Germany; ^6^ Epidemiology and Biostatistics Division, School of Public Health, University of the Witwatersrand, Johannesburg, South Africa; ^7^ Public Health Department, Centre National de Recherche et de Formation sur le Paludisme (CNRFP), Ouagadougou, Burkina Faso; ^8^ Basic Science Laboratory, US Army Medical Research Directorate-Africa/Kenya Medical Research Institute, Kisumu, Kenya; ^9^ Kenya Medical Research Institute, Centre for Clinical Research, Nairobi, Kenya; ^10^ Groupe de Recherche Action en Santé (GRAS), Ouagadougou, Burkina Faso; ^11^ Centre d’Infectiologie Charles Mérieux-Mali, Bamako, Mali; ^12^ Cambridge Institute for Medical Research, University of Cambridge, Cambridge, United Kingdom

**Keywords:** *Plasmodium falciparum* malaria, PF3D7_1136200, ARMA, antigen diversity, IgG and IgM antibodies, vaccines, protein microarray

## Abstract

**Introduction:**

Detailed analyses of genetic diversity, antigenic variability, protein localization and immunological responses are vital for the prioritization of novel malaria vaccine candidates. Comprehensive approaches to determine the most appropriate antigen variants needed to provide broad protection are challenging and consequently rarely undertaken.

**Methods:**

Here, we characterized PF3D7_1136200, which we named Asparagine-Rich Merozoite Antigen (ARMA) based on the analysis of its sequence, localization and immunogenicity. We analyzed IgG and IgM responses against the common variants of ARMA in independent prospective cohort studies in Burkina Faso (N = 228), Kenya (N = 252) and Mali (N = 195) using a custom microarray, Div-KILCHIP.

**Results:**

We found a marked population structure between parasites from Africa and Asia. African isolates shared 34 common haplotypes, including a dominant pair although the overall selection pressure was directional (Tajima’s D = -2.57; Fu and Li’s F = -9.69; P < 0.02). ARMA was localized to the merozoite surface, IgG antibodies induced Fc-mediated degranulation of natural killer cells and strongly inhibited parasite growth in vitro. We found profound serological diversity, but IgG and IgM responses were highly correlated and a hierarchical clustering analysis identified only three major serogroups. Protective IgG and IgM antibodies appeared to target both cross-reactive and distinct epitopes across variants. However, combinations of IgG and IgM antibodies against selected variants were associated with complete protection against clinical episodes of malaria.

**Discussion:**

Our systematic strategy exploits genomic data to deduce the handful of antigen variants with the strongest potential to induce broad protection and may be broadly applicable to other complex pathogens for which effective vaccines remain elusive.

## Introduction

Malaria remains a major public health concern with significant morbidity and mortality (1, 2). Current control tools target vectors, prevent infection, expedite diagnosis and optimize case management. In spite of this, there were still 240 million cases and 602 000 deaths due to malaria worldwide in 2020 (1). The highest disease burden is caused by the most deadly species, *Plasmodium falciparum*, and occurs primarily in low-income countries in sub-Saharan Africa (1). Additional tools are urgently needed to eliminate malaria and highly effective vaccines could be transformative (3). The only malaria vaccine that has been approved by the World Health Organization, RTS,S, induces modest protection that wanes rapidly and has been associated with a rebound of cases (4, 5). Studies on other parasite antigens remain an urgent priority and may increase the likelihood of more efficacious vaccines.

The majority of the genes that encode potential malaria vaccine candidates are polymorphic and induce protection against some but not all parasite strains (6–9). Nevertheless, the impact of antigen diversity on immunity is rarely investigated systematically. Detailed analyses of the large number of antigen variants that may be encoded at a single *P. falciparum* locus are challenging. High-throughput protein microarrays can be designed to analyze strain-specific antibodies (10, 11), but have mainly been utilized to down-select individual antigens that are associated with protection (12–16). Robust approaches to tackle antigen diversity are imperative for the next generation of malaria vaccines.

Technological advances have fast-tracked the discovery of novel targets of protective immunity and can be harnessed to address the challenges posed by antigen diversity (12, 17–22). We provide a contemporary pipeline using the recently proposed vaccine candidate, PF3D7_1136200, which we named Asparagine-Rich Merozoite Antigen (ARMA) based on the nature of its amino acid sequence, localization and immunogenicity. The protein is conserved across *Plasmodium* species and thought to have a predicted signal peptide as well as a glycosylphosphatidylinositol (GPI) modification site (23). Transcriptomic studies indicate that it is highly expressed late in the erythrocytic cycle, but its function remains uncharacterized (24, 25). Of thirty-six antigens analyzed in a prospective cohort study in Kenya, antibodies against ARMA were the most strongly associated with protection (22).

We began by leveraging the Malaria Genomic Epidemiology Network (MalariaGEN) consortium’s pf3k dataset to conduct the first large-scale assessment of the diversity of the *arma* gene. We compared population structure between parasite isolates from West and Central Africa (WCA) and those from South-East Asia (SEA), identified dominant haplotypes and the most common genetic variants at this locus. We then expressed the corresponding full-length ARMA recombinant proteins for immunological assays and characterized its subcellular localization. Finally, we investigated the impact of antigenic diversity on protective immunity by analyzing i) functional antibodies targeting the dominant variant antigens of ARMA, and ii) associations between IgG and IgM antibodies against multiple variants of ARMA and the risk of developing clinical episodes of malaria in independent prospective cohort studies in African children. We deduce the handful of ARMA variants that have the strongest potential to induce broad protective immunity and propose an analytical strategy that can be applied to a wide variety of infectious diseases.

## Materials and methods

### Computational analyses

We used the variant call format (VCF) file of chromosome 11 from MalariaGEN consortium’s open access Pf3k pilot data, version 5.1 (https://www.malariagen.net/data/pf3k-5). We used a suite of computational programs to extract and analyze *arma* variants from the Pf3k dataset based on its coordinates in the 3D7 genome. The VCF file was filtered based on the read depth (60,000 – 260,000) and mapping quality (59.9 – 60.02) to mask polymorphisms at regions that had low read coverage using vcfR tool (26). We further filtered for low quality variants and samples with PLINK (27). Variants and samples with missing call frequencies > 10% and 5%, respectively, were removed.

We used Wright’s fixation index F_ST_ (28) to estimate population structure between sampling locations. We generated a matrix of pairwise F_ST_ values and applied a hierarchical clustering algorithm to group populations that are interbreeding. Several parameters were calculated to measure the level of the genetic diversity within each parasite population including the total number of non-synonymous polymorphic sites, total haplotype number, nucleotide diversity, haplotype diversity and minor allele frequencies along the gene. To evaluate overall and region-specific selection pressure in each parasite population, Tajima’s D (29) and Fu and Li’s F (30) indexes were calculated on the full-length *arma* gene and by a sliding window manner (window length = 20 nucleotides, step size = 8 nucleotides). A network analysis based on the integer neighbor joining method in PopART (31) was used to analyze the distribution of unique haplotypes by country. This method constructed a network tree based on the Hamming distance matrix between haplotypes using the neighbor-joining method coupled with the integer linear programming (31).

Orthologous protein sequences of ARMA that were available in PlasmoDB (https://plasmodb.org/plasmo/app/record/gene/PF3D7_1136200#category:evolutionary-biology) were used for conservation analysis. ClustalW multiple alignment was first performed with 45 sequences from 16 different *P. falciparum* strains and 20 other *Plasmodium* species. The output was then used to conduct sequence logo and Shannon entropy (32) analyses to evaluate the amino acid conservation across *Plasmodium* species. The aligned orthologous sequences were also used to draw a phylogenetic tree *via* the unweighted pair group method with arithmetic mean in MEGA (33). We selected *Vinckeia* species as an outgroup in relative time tree analysis.

### Recombinant protein expression and western blot

The top 2 dominant variants were expressed as recombinant proteins using the Expi293 expression system (Invitrogen) as previously described (21) Briefly, the sequences of the protein variants were designed by excluding the predicted signal peptide and GPI modification site and by replacing serine or threonine residues by alanine at all N-glycosylation sites. The DNA molecules that encode these designed proteins were made by gene synthesis by GeneartAG as codon-optimized sequences flanked by NheI and BamHI restriction sites for expression in the mammalian system. The genes were subsequently sub-cloned into the pTT28 expression vector (obtained from National Research Council Canada Biotechnology Research Institute) by restriction digest. The vector contained N-terminal human alkaline phosphatase signal peptide and C-terminal octahistidine tag (His8G). Following cloning, the expression constructs were used to transfect 2 x 10^6^ cells/ml Expi293F culture at > 95% viability to obtain final 1 µg/ml DNA concentration using ExpiFectamine 293 transfection kit (Gibco). The enhancers were added 16 – 18 hours after transfection and the recombinant proteins were harvested 2 to 4 days later in the supernatant.

The full-length C-terminal his tagged proteins were purified using the Ni-NTA purification system in native conditions (QIAGEN). The pre-charged Ni-NTA agarose with Ni^2+^ ions was added to culture supernatants and incubated at 4°C to allow proteins to bind. The bound resins were then transferred to polypropylene columns for wash and elution using imidazole-containing buffers in gravidity-flow chromatography. The BCA protein assay kit was used (Thermo Scientific) to quantify the purified proteins before running sodium dodecyl sulfate–polyacrylamide gel electrophoresis (SDS-PAGE) and western blot. SDS-PAGE was performed with 10 µl of eluates in reducing conditions and proteins were transferred onto methanol-activated polyvinylidene fluoride (PVDF) membrane for 1 hour at 80 V for western blot. Membrane was blocked with 5% Blotto, non-fat dry milk (ChemCruz) in TBS and HRP-conjugated anti-histidine antibodies were used for detection.

### Antigen enzyme-linked immunosorbent assay

To test our recombinant protein variants for immunogenicity, a 3-day ELISA protocol was performed as previously described (34). Briefly, 1 µg/ml of our proteins in sodium carbonate and bicarbonate buffer was coated in 96-well plates (100 µl/well) for an overnight incubation at 4° C. Thereafter, plates were washed with phosphate-buffered saline (PBS) at 0.05% tween 20 (PBST) before blocking with 1% skimmed milk in PBST (block buffer, 200 µl/well) for 5 hours. After wash, diluted plasmas from Kenyan children in block buffer (1:1000, 100 µl/well) were added for another overnight incubation at 4°C. On the following day, plates were incubated with HRP-conjugated rabbit anti-human IgG diluted in block buffer (1:5000, 100 µl/well) for 3 hours after wash. To develop our reactions, a final wash of the plates was performed followed by an incubation with the substrate, o-Phenylenediamine dihydrochloride-based solution (200 µl/well), for 25 minutes. Finally, 2M H_2_SO_4_ was added to stop the reaction and the optic density was immediately measured at 492 nm.

### Merozoite extract western blot

Merozoite protein extracts were prepared by adding merozoites to SDS sample buffer supplemented with Dithiothreitol. The suspension was heat-denatured for 10 min at 80°C before loading it on 10% SDS polyacrylamide gel. After running at 120 V, the protein extract was transferred onto an activated PVDF membrane at 125 mA for 2 hours. The membrane was blocked afterwards with 3% skimmed milk in PBST overnight at 4°C. After wash, the membrane was probed with diluted rabbit antisera (anti-V1 and anti-V2) in blocking buffer (20 mg/ml) for 90 minutes at room temperature while rotating. The membrane was washed with PBST for 6 minutes before adding secondary antibodies (alkaline phosphatase-conjugated goat anti-rabbit IgG diluted in blocking buffer, 1:80,000) for 2-hour incubation at room temperature while rotating. After final wash, bound antibodies were detected by adding 5-bromo-4-chloro-3-indolyl phosphate/nitro blue tetrazolium-buffered substrate and the reactions were stopped in cold ddH_2_O.

### Immunofluorescence assay

A synchronized *P. falciparum* culture at 4 – 5% parasitemia was incubated with 10 μM E64 until schizont maturation. Thereafter, the culture medium was removed and the infected RBC pellet was washed with PBS. For fixation, the pellet was resuspended in 4% paraformaldehyde/0.0075% glutaraldehyde in PBS and incubated while shaking at 37°C for 1 hour. For permeabilization, the infected red blood cells were incubated in 1 ml of 125 mM glycine/0.05% Triton-X-100 in PBS while shaking at room temperature for 30 minutes. After washing, the pellet was blocked with 3% bovine serum albumin (BSA) in PBS overnight at 4°C. On the next day, the suspension was split and two parts were resuspended in 100 µl of diluted test sample (either anti-sera or pre-immune sera) and anti-MSP1 (full-length) antibodies in 3% BSA/PBS/0.05% Triton-X-100 (1:100) and incubated overnight while rotating at 4°C. One part without antibodies was used as a staining control. On the last day, the samples were washed with PBS before adding the secondary antibodies (AlexaFluor 488-conjudated goat anti-mouse IgG and Cy5-conjugated goat anti-rabbit IgG diluted, 1:500, in 3% BSA/PBS). After incubation for 1 hour while rotating in the dark at room temperature, the samples were washed and Hoechst 33258 (1 μg/ml in PBS) was added. Finally, the pellets were resuspended in 100 µl of PBS before mounting on microscope slides coated with concanavalin A. Images were acquired using a confocal microscope (Nikon Eclipse Ti) and analyzed by Fiji.

### Functional antibody assays

#### NK cell degranulation assay

This experiment was performed using a protocol that was recently developed in our laboratory (35). Briefly, following coating 30 ng/ml recombinant proteins; V1, V2 and MSP1 (control), in 96-well culture plate at 4°C overnight, the wells were washed with PBS and blocked for 2 hours at 37° C with 1% casein in PBS. Diluted heat-inactivated (1:20) MIG, PHIS and naïve serum (negative control) were added in the coated plate. After incubation for 5 hours at 37°C, freshly isolated NK cells from peripheral blood mononuclear cells from malaria-naive individuals were added (2.0×10^3^ NK cells/well, purity > 90%) as well as monoclonal anti-human CD107a – Phycoerythrin (1:50, surface marker of degranulated NK cells), brefeldin A and monensin (5 μg/ml). The plate was incubated for 18 hours at 37° C in 5% CO_2_ thereafter. Finally, the NK cells were washed, centrifuged, resuspended in Fluorescence-Activated Cell Sorting buffer (PBS, 1% BSA, 1mM ethylenediaminetetraacetic acid and 0.1% sodium azide) and stored at 4°C until acquisition. Their viability was checked by staining with a fixable viability dye eFluor^™^ 520 for 10 minutes at 4° C. The BD FACSCaliburII high-throughput system was used for acquisition and data was analyzed in Flow.Jo V10.

#### Growth inhibition assay

To perform this assay, an ultra-synchronized *P. falciparum* culture (Pf3D7 strain) adjusted to 0.5% parasitemia at 1% hematocrit was used. Serial dilutions of rabbit antisera (anti-V1, anti-V2), pre-immune sera, MIG and anti-AMA1 DiCo antibodies (10 – 2.5 mg/ml) were prepared using the culture medium. We added 10 µl of these antibodies to 40 µl of parasite culture in triplicates in 96-well U-bottomed plates. To prepare parasite growth controls, 10 µl of antibody-free medium were also added in separate culture wells and processed similarly along with the samples. The outer wells of the plates were filled with 50 µl of culture medium for humidification purposes. After the first replication cycle (48 hours), 10 µl of fresh culture medium were added to every well to protect parasites from starvation. At the end of the second replication (96 hours), plates were centrifuged at 1800 rpm for 3 minutes to discard the medium. The pellets were washed with sterile PBS and the parasites were stained with SYBR Green in dark for 30 minutes. Plates were washed later before fixing the cells in 2% Paraformaldehyde/PBS and parasite density were measured by flow cytometry. The growth inhibition rate was calculated as follows:


$$\text{Growth inhibition rate (\%)}=100-100\times \frac{\text{Paresitemia of treated parasites}}{\text{Mean parasitemia of untreated (control) parasites}}$$

#### Cohort studies

We harnessed retrospectively cryopreserved (-80° C) archived samples and clinical data collected from three independent cohort studies in West and East Africa along local institutional and national scientific and ethical guidelines. We used all available samples that were collected during initial visits of each participating study. Although the transmission profile of malaria in the three study sites was different, the design of the prospective cohort study was similar. Participants included children aged between 0 and 5 years.

The first study was conducted in Kombewa Division, formerly in Kisumu District in western Kenya. Malaria transmission is hyperendemic in the area with two peaks corresponding to long (March to June) and short (November to December) rainy seasons (36). The monthly incidence of malaria episodes ranged between 20 and 55% (36). The entomological inoculation rate (EIR) was estimated at 0.65 – 0.79 infectious mosquito bites per person per night (37). This study was a randomized phase IIb clinical trial of an MSP1_42_ vaccine that was not protective. Participants were followed over 6 months through bi-weekly active visits from 2004 to 2005 and passive unscheduled visits. A malaria episode was defined as the presence of parasitaemia by microscopy (≥ 50,000 parasites/µl) and fever (≥ 37.5°C).

The second study was conducted in the Saponé health district, in Balonghin, 40 km south of Ouagadougou, the capital city of Burkina Faso. Malaria transmission is seasonal and peaks during the rainy season from June to September, when the estimated EIR reaches 44.4 infective bites per person per month (38). The study was originally conducted to evaluate malaria epidemiology in preparation for drug and vaccine clinical trials. Participants were recruited in June 2007 and followed fortnightly over 12 months through active and passive visits (38). The definition of a malaria episode was the presence of microscopic parasitaemia (≥ 5000 parasites/μl in the high season and ≥ 2500 parasites/μl in the low season) and fever (≥ 37.5°C).

The third study was conducted in Bandiagara (Mali), a Savannah-Sahel area in northeast Mali. Malaria transmission is highly seasonal from June to December and becomes intense in August – September during the rainy season when the EIR is estimated at 60 infective mosquito bites per person per month (39, 40). Children aged between 1 and 5 years experience an average of 2 malaria episodes each year (40). The aim of this longitudinal cohort study was to validate putative asexual blood stage antigens as vaccine candidates. Participants were enrolled in June 2008 and monitored weekly through active and passive visits until May 2009. A malaria episode was defined as the presence of parasitaemia by microscopy (2500 parasites/µl) and fever (≥ 37.5°C).

#### Div-KILCHIP v1.0 design and printing

We developed our custom protein microarray as previously described (14) with minor modifications. Our chip was designed to contain 35 proteins, including 27 variants of ARMA and full-length AMA1, MSP1, MSP3, MSRP4, P113, SEG2, SERA3 and SERA7 (used as controls, **Supplementary Methods**); 12 immunoglobulin controls and 13 blanks (printing buffer only). The immunoglobulin controls included 6 fluorochrome-conjugated anti-human IgG and IgA, 3 capture human IgG and 3 commercial rabbit anti-human IgG. Fluorochrome-conjugated immunoglobulins were used as landmarks to help visualize the miniarrays after printing. Capture IgG was used to check the quality of the test serum. The commercial antibodies were used to validate our sample processing because they bind the secondary antibodies. We used 24-miniarray nitrocellulose slides (ONCYTE SuperNOVA, GraceBio). Proteins were diluted to 250 µg/ml in the printing buffer (50% glycerol and 1% Triton X-100 in distilled water) prior to printing. The printing was performed by non-contact double dropping of 100 pl of diluted proteins at 23°C and 60% humidity using the Marathon micro-arrayer (ArrayJet advance) that was endowed with the Inkjet printing system (ArrayJet advance). Proteins and controls were spotted in triplicate, generating 180 spots per miniarray. After printing, slides were kept in the printer to dry for 1 hour and stored at 4° C until processing.

#### Div-KILCHIP processing

Before processing, microarray slides were pre-scanned to check the quality of the miniarrays after one-month storage at 4° C. Four slides were assembled into a 4 x 24 hybridization cassette (Arrayit Corporation, ARYC), allowing to process one sample per miniarray, 24 samples per slide and 96 samples per cassette (equivalent to a 96-well ELISA plate). An MIG titration was performed per sample batch (run) using 11 concentrations ranging between 533 and 0.009 µg/ml to check the stability of our experiments. In total, 3 PHIS, 22 European naïve sera and 38 blanks were run on the slides along with the samples. We began by washing the wells (miniarrays) twice with 200 µl of 0.1% tween 20 in 1X 4-(2-hydroxyethyl)-1-piperazineethanesulfonic acid (HEPES) buffer saline (HBS: 1.4 M NaCl, 50 mM KCl, 20 mM CaCl_2_, 10 mM MgCl_2_ and 100 mM HEPES) (wash buffer 1) and once with 200 µl of 1X HBS (wash buffer 2) for 5 minutes while shaking at 300 rpm. After 3 washes (2 times with wash buffer 1 and one time with wash buffer 2), slides were blocked with 200 µl of 2% IgG-free bovine serum albumin in wash buffer 1 (Block and serum dilution buffer) per well for 2 hours at room temperature while rotating in dark. We added 150 µl of diluted serum samples in a block buffer (1:400) per well after 3 washes and incubated the slides overnight at 4° C while rotating in the dark. On the next day; the two secondary antibodies, Alexa Fluor^®^ 647-conjugated donkey anti-human F_Cγ_ (IgG) fragment (Jackson ImmunoResearch) and goat Alexa Fluor^®^ 555-conjugated anti-human IgM (SouthernBiotech), were mixed and diluted in block buffer (1:800). Following blocking, slides were washed 3 times before adding 150 µl of diluted secondary antibodies per well for 3-hour incubation at room temperature while rotating in dark. After this step, slides were washed 3 times before we disassembled them from the hybridization cassette for final rinse in distilled water using Coplin jars. Slides were dried by moderate centrifugation (300 g) for 5 minutes using a CombiSlide adapter (Eppendorf). Finally, slides were scanned using GenePix 4000B Microarray Scanner (Molecular Devices) coupled to GenePix Pro 7 Microarray Acquisition and Analysis Software, which enabled data extraction from the slide images as MFI. IgG and IgM reactivities were detected simultaneously using different channels at 635 and 532 nm, respectively.

#### Data analysis

For each protein, we used the average of the median MFI values of the 3 spots statistical analysis after subtracting background intensities. Data quality control was performed using R v3.6.1 and included evaluation of background intensities, buffer signals, replicate correlation, and negative and positive controls (**Supplementary Figures 9**–**14**). In total, we used 22 serum samples from naive Europeans as negative controls and performed a principal component analysis on the raw data that showed a clear separation of these samples and the blank from the rest of individuals analyzed (**Supplementary Figure 14**). Outliers (5.7%) corresponding to degraded slide images (**Supplementary Figure 11**) were removed from the final data. To reduce batch effect and systematic variations, we normalized the data using *ComBat* function (*SVA* package in R) and *VSN* methods (41, 42), respectively, as previously reported (**Supplementary Figure 15**, **16**) (14). The univariate analysis and data visualization were performed using *stats*. For multivariable analyses of the magnitude of the MFI responses *ComplexHeatmap*, *FactoMiner* and *factoextra* packages were used. Population-level variations of antibody reactivities were estimated by hierarchical clustering of the Euclidean distances between individual MFI values. Hierarchical clustering of Spearman correlation matrix was performed to evaluate the relatedness between antigen variants in terms of antibody responses. To measure seropositivity and investigate association with protection, we harnessed the “maximize_metric” model built in *cutpointr* R package to classify antibodies responses into two categories (positive and negative) based on malaria status and study sites as covariate. The *cutpointr* model defines a cutoff from the distribution of normalized antibody MFIs for each antigen above which individuals are considered seropositive. A modified Poisson regression model based on antibody response classes and clinical episodes of malaria was fitted in which age was considered as a covariate as previously used (43, 44). This analysis was performed using a suite of R packages including *sandwich*, geepack and *lmtest* and estimated the risk ratio of malaria episodes during follow-up, 95% confidence interval and P-value for each antigen variant. To perform the combinatory analysis of IgG and IgM, we first calculated cross-seropositivity scores (1 or 0) which referred to the *cutpointr* classifications for different antibody isotypes concurrently within the same individual. Cross-seropositivity score of 1 for a sample means a positive *cutpointr* classification of all antibodies of the combination and 0 if at least one antibody is classified as negative. The cross-seropositivity variable was then used in the modified Poisson regression model to analyze protective association. Data visualization was performed using *ggplot2* and *ggpubr* packages.

## Results

### Marked differences in the population structure of *arma* in Africa compared to Asia

**Figure 1:**
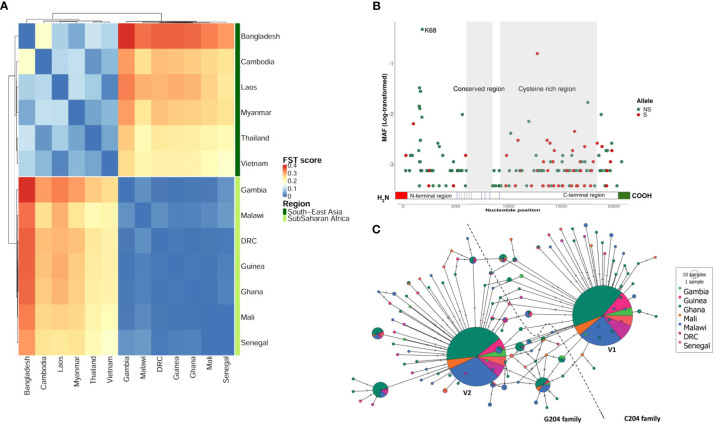
Sequence diversity of ARMA. **(A)** Population differentiation between West and Central African and South-East Asian parasite populations. The heatmap shows all FST values after a pairwise analysis (N= 2,317). **(B)** Minor allele frequencies along the gene. Each dot represents a unique allele which is either synonymous (S) or nonsynonymous (SN). The minor allele frequencies (MAF) are shown as log-transformed values. Only two alleles had a frequency above 10%. The conserved region contained no SNPs. N68K is the most frequent polymorphism observed. **(C)** Haplotype network based on the hamming distance between gene variants in West and Central Africa. Each pie-chart represents a unique haplotype based on nonsynonymous SNPs. Colors code for geographical locations of the samples. Pie-chart size is proportional to haplotype frequency in the population. The lines (branches) between haplotypes represent ancestral relationships, i.e., the degree of relatedness. The two equally dominant haplotypes (largest pie-charts) differ by a single nucleotide at position 204. The dashed line separates the C204 and G204 families. The haplotype found in the *P. falciparum* 3D7 strain belongs to the C204 family. DRC, Democratic Republic of Congo.

We first analyzed 2,317 nucleotide sequences of field *P. falciparum* isolates from 13 countries in WCA and SEA, including Democratic Republic of Congo (DRC) (*n* = 106), The Gambia (*n* = 58), Ghana (*n* = 569), Guinea (*n* = 100), Malawi (*n* = 363), Mali (*n* = 68), Senegal (*n* = 69), Bangladesh (*n* = 44), Cambodia (*n* = 554), Laos (*n* = 85), Myanmar (*n* = 60), Thailand (*n* = 145) and Vietnam (*n* = 65). We identified in total 208 bi-allelic SNPs, 4 multi-allelic SNPs and 13 indels within the 5 exons of the *arma* gene after variant filtering. Repartitioning these SNPs by region revealed 199 in WCA and 26 in SEA. Only 13/212 SNPs were shared between both regions and there was no correlation in the frequencies of these shared alleles between regions (Pearson’s R = 0.25, *P = 0*.*41*). This indicated a marked population structure and was confirmed by high F_ST_ values (0.18 - 0.38, **Figure 1A**). For instance, the most abundant allele at amino acid position 204 had a minor allele frequency (MAF) of 47.5% in WCA but was markedly lower at 5.3% in SEA. The pairwise F_ST_ index ranged from 0.0005 to 0.03 within WCA and from 0.015 to 0.181 in SEA (**Figure 1A**). We observed a limited subpopulation structure between the West and one central African country (Malawi, F_ST_ = 0.01 – 0.03) but not the other (DRC, F_ST_ = 0.0009 – 0.005). The sliding window nucleotide diversity (π scores) and Tajima’s D were consistently higher in SEA than WCA along the entire length of the gene with the exception of the C-terminal where π scores were comparable (**Supplementary Figure 1**).

### Significant *arma* genetic diversity in Africa masks a pair of dominant haplotypes

Given the pronounced differences in population structure between Africa and Asia, and because Africa accounts for > 95% of the global malaria burden (1), we focused further analyses on African sequence data (seven countries, *n* = 1,333). We found 159 unique SNPs within the 5 exons of the gene; 48 were synonymous and 111 were nonsynonymous. We retained the latter for haplotype analysis following data phasing. We found 125 unique haplotypes with π scores and haplotype diversity ranging between 4.33x10^-3^ and 4.82x10^-3^ and between 0.65 and 0.70, respectively (**Supplementary Table 1**). The overall selection pressure was directional with significant negative Tajima’s D and Fu and Li’s F values (Tajima’s D = -2.57; Fu and Li’s F = -9.69; *P<* 0.02). The levels of individual genetic diversity and selection pressure were similar across the seven African countries studied. We found that 30% of SNPs (47/159) clustered within a 609 nucleotide-long segment of the N-terminal region (1 – 609), 87% (41/47) of which were non-synonymous (**Figure 1B**). The most dominant SNP (position 204) was also located in the same region which showed highest levels of π score, haplotype diversity and Tajima’s D index (**Supplementary Figure 1**). The nonsynonymous MAF/synonymous MAF ratio was 1.6 for the entire gene, but reached up to 8.3 for the N-terminal region alone compared to 0.3 for the C-terminus (919 – 1809). We identified a highly conserved region (609 – 848) that did not have any mutations in the 1,333 samples analyzed. Based on a multiple alignment of 45 orthologous sequences from 16 P*. falciparum* strains and 20 other *Plasmodium* species, we found that the C-terminal region is conserved across the *Plasmodium* genus (average Shannon entropy value was 0.43, **Supplementary Figure 2A**). Interestingly, this region contained up to 9 cysteine residues that are present in all species studied (**Supplementary Figure 2B**).

To determine how the different variants were related and distributed geographically, we performed a haplotype network analysis based on non-synonymous SNPs (**Figure 1C**). Singletons were highly abundant (91/125 haplotypes, 72.8%) but present in only a minority (6.8%) of samples. In contrast, two of the remaining 34 haplotypes were found in the majority (79%) of parasite isolates. This pair of dominant variants differed by a single SNP at position 204 where a transversion mutation (C by G) results in the substitution of asparagine (N) by lysine (K) at position 68 of the protein. We therefore defined two haplotype families C204 (represented in the 3D7 strain) and G204, and found that they were equally distributed (52 vs. 48%, respectively) in the African countries studied (**Figure 1C**). The G204 family showed a relatively higher level of sequence diversity and included 1.4-fold more unique haplotypes (more ancestral branches in the network, **Figure 1C**) than C204. Phylogenetic and relative divergence time analyses of the orthologous sequences suggested that K68 is the ancestral allele and N68 may have been fixed in the *Laverania* species 0.07 million years ago (**Supplementary Figure 3**)

### Localization of ARMA to the merozoite surface

**Figure 2:**
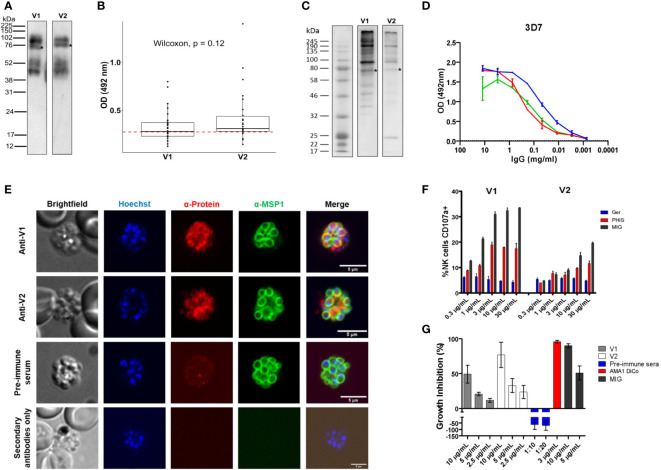
Subcellular localization of ARMA and induction of functional antibodies. **(A)** Western blot of recombinant proteins after purification. Four bands were detected using anti-histidine antibodies. Asterisks indicate the full-length proteins (75 KDa). **(B)** Immunogenicity of recombinant antigen variants V1 and V2 in Kenyan children (*n* = 30). The red dotted line represents the threshold of seropositivity which was defined as the mean of optic density (OD) + 2 standard deviations in malaria-naïve controls. **(C)** Merozoite extract western blot. Multiple bands were detected using rabbit antisera (primary antibodies) and anti-human antibodies (secondary antibodies). Asterisks show full-length proteins. **(D)** Merozoite ELISA using V1/V2 antisera shows specific dose-dependent responses in the 3D7 strain. Antigen-specific antibodies against apical membrane antigen 1 (AMA1) served as a positive control. **(E)** Immunofluorescent staining showing the colocalization of ARMA with MSP1 on the merozoite surface. Late schizont stages were double stained with rabbit antisera (anti-V1 or anti-V2) and mouse anti-MSP1 (full-length) in immunofluorescence assay. Rabbit preimmune sera were used to assess non-specific binding. The merozoite nuclei were stained with Hoechst. Incubation of parasites with only anti-rabbit antibodies excluded non-specific binding of secondary reagents. **(F)** Dose-dependent NK cell degranulation with antibodies against variants V1 and V2. Ger: Malaria-naive serum from German donors. **(G)** Dose-dependent growth inhibition activities of rabbit anti-sera (anti-V1 and anti-V2) in three independent assays. Rabbit preimmune sera, anti-AMA1 DiCo and MIG were used as controls.

We expressed the two dominant variants of ARMA (V1 and V2) as full-length recombinant proteins. They were detected in Western blots as four bands: the full-length proteins at 75 KDa and fragments of approximately 40, 52 and 100 KDa, respectively (**Figure 2A**) (45). We tested the immunogenicity of the recombinant proteins in plasma samples from children aged between 1 and 5 years (n = 30) from Kisumu, Kenya. Antibody reactivity against both variants was comparable in enzyme-linked immunosorbent assays (ELISAs, Wilcoxon, *P* = 0.12, **Figure 2B**). In Western blots loaded with merozoite extracts, V1 and V2 rabbit antisera bound to multiple bands of approximately 190, 130, 100 and 75 KDa, respectively, corresponding to the intact full-length proteins (**Figure 2C**). In ELISAs, both antisera recognized merozoites of two different *P. falciparum* strains, 3D7 (C204, **Figure 2D**) and Dd2 (G204) in a dose-dependent manner and at comparable levels. The majority of parasite ligands belonging to the merozoite surface protein (MSP) family, such as MSP1, contain GPI anchors and cysteine-rich domains (23). We conducted immunofluorescence assays (IFAs) to test whether ARMA localized to the merozoite surface. Both V1 and V2 antisera labeled the merozoite surface, partially overlapping the signal detected from anti-MSP1 antibodies (**Figure 2E**).

### ARMA antibodies mediate Fc-dependent and independent parasite killing

We then tested whether antibodies targeting ARMA mediated parasite killing or the inhibition of red cell invasion through Fc-dependent and independent mechanisms, respectively. Antibodies against ARMA neither bound to complement C1q (**Supplementary Figure 4A–D** (46),), nor induced the release of reactive oxygen species from neutrophils (**Supplementary Figure 4E–H** (47, 48),). However, anti-ARMA antibodies induced the dose-dependent degranulation of natural killer cells, albeit in a strain-specific fashion (**Figure 2F**). Similarly, anti-ARMA antibodies inhibited parasite growth in the Fc-independent two-cycle assays. We observed a modest degree of strain-specificity in growth inhibition but the differences were not statistically significant (**Figure 2G**).

### Profound IgG and IgM serological diversity is restricted to just three serogroups

To better understand the variation in antibodies against ARMA variants, we analyzed the protein sequences of all the haplotypes (n = 34) that were commonly observed in African isolates. Removal of the predicted signal peptides and GPI modification sites resulted in 27 haplotypes (including the two dominant ones, **Supplementary Table 2**). These contained 18 polymorphic sites (10 in the N-terminal region) and 1 - 4 pairwise amino acid differences. All 27 variants were expressed as C-terminal octahistidine-tagged proteins in Expi293F cells and purified under native conditions (21). As previously observed for V1 and V2, multiple bands were detected for each protein in Western blots (**Figure 2A**). All proteins were tested for immunogenicity by ELISA before printing on our custom microarray, Div-KILCHIP v1.0. Native recombinant proteins reacted with pooled hyperimmune immune sera (PHIS) from malaria-exposed individuals in Kenya but not with serum from malaria-naive Europeans (**Supplementary Figure 5B**). Reactivity was retained following heat denaturation and suggested that the proteins contained linear epitopes (**Supplementary Figure 5C**).

**Figure 3:**
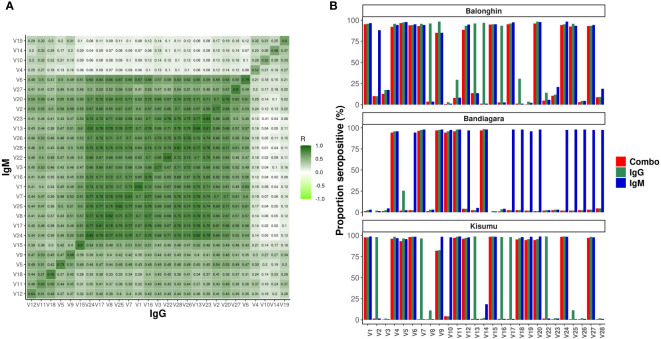
Profound diversity of IgG and IgM antibodies to variants of ARMA in Balonghin, Burkina Faso, Bandiagara, Mali and Kisumu, Kenya. **(A)** Correlation of normalized MFIs for IgG and IgM against 27 variants of ARMA. Pairwise correlation between IgG and IgM reactivities assessed using Spearman’s R **(B)** Seroprevalence ranged between 0.8 - 98.8%, but was comparable between IgG vs. IgM; *P* = 0.98, Wilcoxon test. The levels of IgM antibodies were higher than IgG, *P<* 0.001, Wilcoxon test. Combo refers to samples with detectable levels of both IgG and IGM against the same antigen variant.

We measured antibodies in archived plasmas from prospective cohort studies in Balonghin, Burkina Faso, Bandiagara, Mali and Kisumu, Kenya (*n* = 715). Data from 675 children (94.3%) met the protein microarray quality control criteria (**Supplementary Methods**) and are summarized in **Table 1**. We detected IgG and IgM antibodies against all 27 variants. Although only a maximum of four pairwise amino acid differences was detected between protein variants (**Table 1**), their impact on antibody diversity was profound. Seropositivity ranged between 0.8 - 98.8% for different variants but was majorly similar for IgG and IgM (**Figure 3B**) across the three sites. Antibody levels were generally higher for IgM compared to IgG (*P<* 0.001, Wilcoxon test, **Figure 3B**). The levels of isotype antibodies against the same variants were strongly correlated (*R = 0.59 – 0.84, P< 0.001*), particularly for 15/27 of them (V24 - V6, and including the dominant V1 and V2, dark green block in **Figure 3B**).

**Table 1:** Participant characteristics.

Characteristics	Malaria episode	No malaria episode	Overall
Age			
Medium ± IQR (month)	28 ± 18	34 ± 20	31 ± 20
0-2 years	143	95	238
2-5 years	212	189	401
Missing	16	20	36
Sampling location			
Balonghin	134	94	228
Bandiagara	91	104	195
Kisumu	146	106	252
Total	371	304	675

IQR, interquartile range.

**Figure 4:**
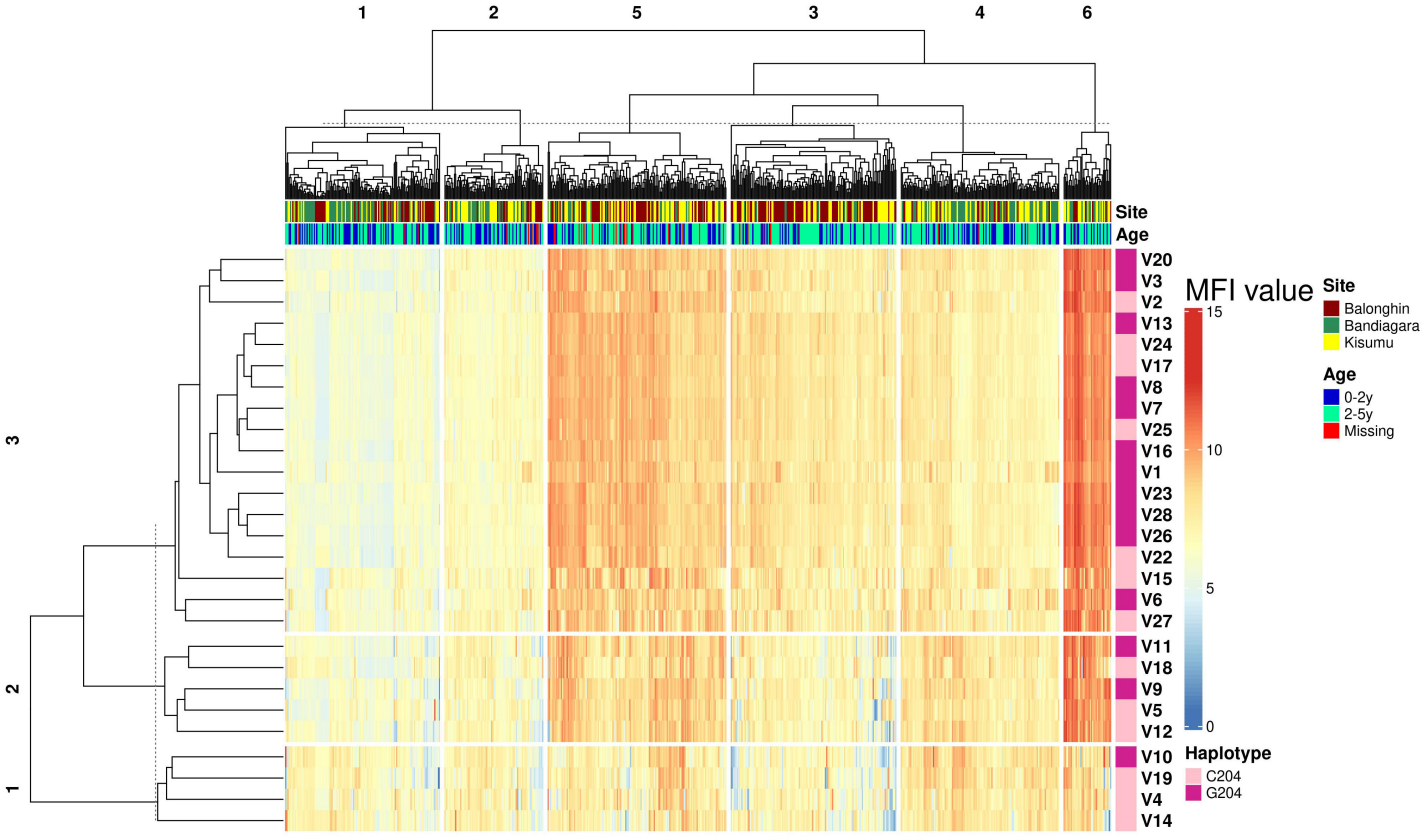
Geographical location is the main determinant of ARMA serogroup. Heatmap combines hierarchical cluster analysis of Spearman’s R (dendrogram on y axis, left) of individual antibody response measurements (MFI values) by geographical location and age group with haplotype diversity (y axis, right). Numbers above dendrograms indicate the ranks of the clusters. C204 and G204 are the two haplotype families of ARMA. Malaria status indicates whether or not a child developed clinical malaria during follow up.

We performed a hierarchical clustering analysis of the pairwise correlation coefficients between antibodies against all variants. Remarkably, these segregated into just three serogroups that were similar for both IgG and IgM (indicated in rows in the heatmap in **Figure 4**). The majority of variants in serogroups 1 and 2 belonged to the C204 haplotype (3/4 and 3/5, respectively), whereas serogroup 3 was dominated by G204 haplotypes (11/18).

### Geographical location is the main determinant of ARMA serogroups

To identify the determinants of serogroups, we conducted a dissimilarity analysis based on the Euclidean distance between individual responses to all variants. We identified six response profiles from 675 individuals across three distinct African populations (P1 – P6, clusters along the columns of the heatmap in **Figure 4**). These response profiles largely reflected geographical locations (**Figure 4**). Profiles P2, P4 and P5 were highly enriched with samples from Balonghin whereas P1 and P3 were dominated by Bandiagara (*P< 0.01*, χ^2^). In contrast, samples from Kisumu were more evenly dispersed across all serogroups. The variation of serogroups by geographical location was confirmed in a principal component analysis (**Supplementary Figure 6**). Age had a modest impact on serogroup profiles; P4 – P6 were significantly enriched with children between 2 and 5 years of age and that had higher antibody responses than their younger counterparts (**Supplementary Figure 7**).

### Protective IgG and IgM target cross-reactive and distinct epitopes within ARMA

**Figure 5:**
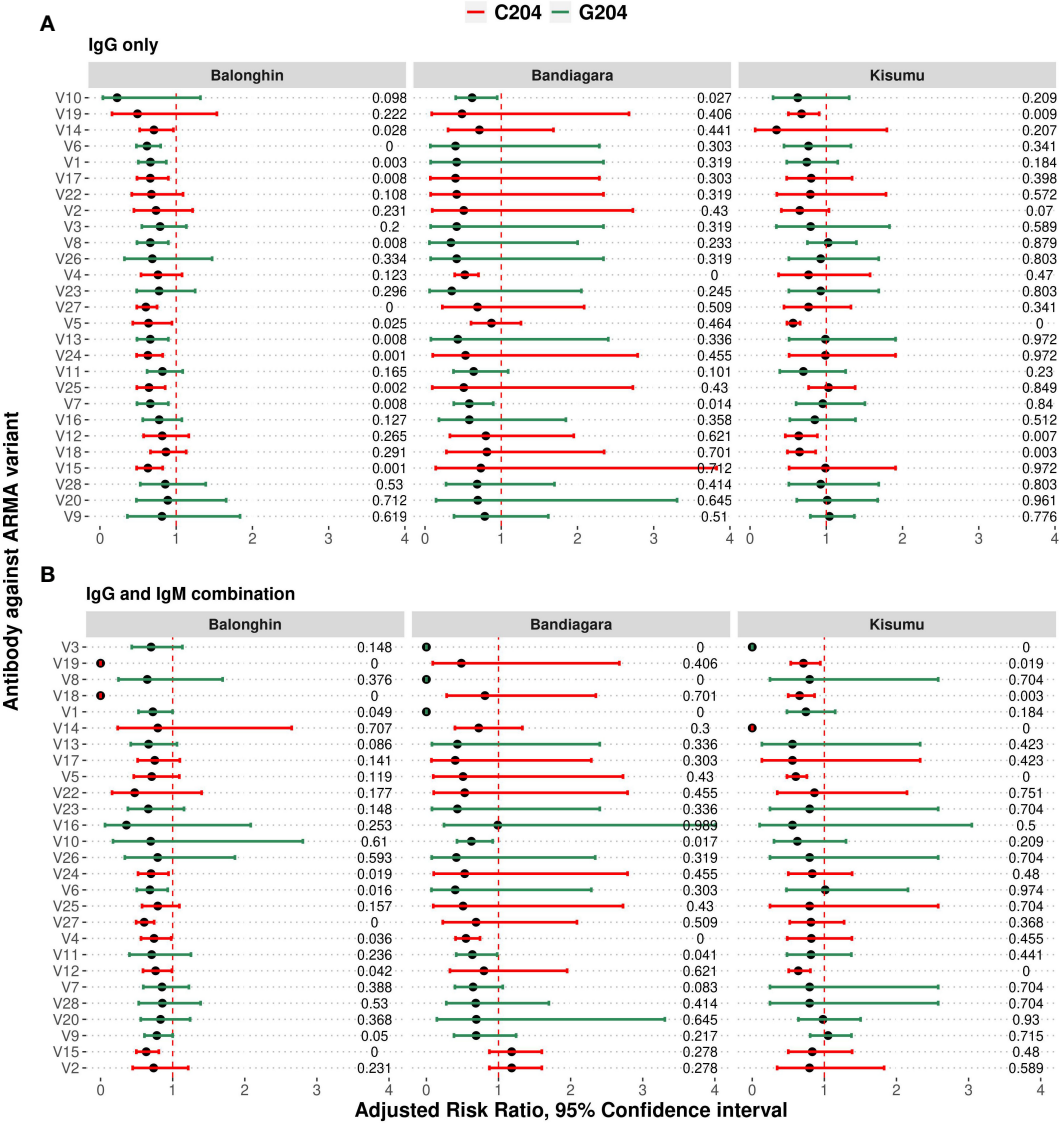
Combinations of IgG and IgM antibodies against selected variants of ARMA are associated with protection against clinical episodes of malaria. **(A)** Associations between IgG antibodies to individual variants and the risk of clinical episodes of malaria. Antibodies against variants containing both C204 and G204 haplotypes were significantly associated with protection in Balonghin and Bandiagara, but only the former in Kisumu. **(B)** Combinations of IgG and IgM antibodies to at least two variants were associated with a 100% reduction in the risk of malaria in all three geographical locations; V18 and V19 in Balonghin, V1, V3 and V8 in Bandiagara, and V3 and V14 in Kisumu. Figures on right represent P-values, red and green represent haplotype families C204 and G204, respectively.

Although we had observed that IgG and IgM responses to ARMA were highly correlated (**Figure 3**), we nevertheless tested whether the same isotype and variant-specific antibodies were associated with protection against clinical episodes of malaria after adjusting for age, and whether these were shared across the geographical locations. In Balonghin, Burkina Faso, IgG antibodies against 12 variants were associated with protection (V14, V6, V1, V17, V8, V27, V5, V13, V24, V25, V7, and V15; **Figure 5A**). At the same location, IgM antibodies to only 7 variants (V18, V12, V1, V27, V6, V2 and V15; **Supplementary Figure 7**) were associated with protection, with only four of these being shared with IgG (V1, V6, V15 and V27). This suggested that potentially protective antibodies targeted both cross-reactive and distinct epitopes between haplotypes. A similar observation was made in Bandiagara, Mali, where IgG and IgM antibodies against 3 (V4, V7, V10; **Figure 5A**) and 5 variants (V19, V10, V12, V4, V24; **Supplementary Figure 7**), respectively, were associated with protection, with only two of these (V4 and V10) being shared between the isotypes. In Kisumu, Kenya, both IgG and IgM antibodies were associated with protection for only 5 variants (**Figure 5A**, **Supplementary Figure 8**), in addition to IgG against three other variants (V12, V18 and V19).

Regardless of isotype, we observed that antibodies against only V12 were associated with protection in all three countries, while those against 5 variants (V5, V7, V18, V19 and V24) were associated with protection in only two (**Figure 5A**, **Supplementary Figure 8**). In total, IgG and IgM antibodies against more than two-thirds of all variants analyzed (19/27) were associated with protection in at least one country, with the majority belonging to the C204 (n = 13/19) haplotype families. Nevertheless, the presence of variant- and isotype-specific responses led us to investigate the effect of combinations of isotype antibodies on protection.

### Combinations of IgG and IgM antibodies against selected variants of ARMA are associated with complete protection against clinical episodes of malaria

We asked whether combinations of IgG and IgM antibodies against individual variants were more strongly associated with protection than either isotype alone. As shown in **Figure 5B**, the number of variants for which significant association of antibodies with protection is observed fell off to 9 in Balonghin but increased in both Bandiagara (6 variants) and Kisumu (6 variants). Interestingly, in every country, we observed that at least two combinations of IgG and IgM against a selected variant was associated with complete protection (V18 and V19 in Balonghin, Burkina Faso; V1, V3 and V8 in Bandiagara, Mali and V3 and V14 in Kisumu, Kenya; **Figure 5**). These variants represented only the C204 haplotype family in Balongin, G204 in Bandiagara and both C204 and G204 (V6, V10 and V23) in Kisumu. Only combination of IgG and IgM against V3 (a G204 haplotype) was associated with full protection against clinical episodes of malaria in two different sites (Bandiagara and Kisumu). By contrast, no association with protection was found with this variant at all for single isotype in all three sites.

## Discussion

Malaria vaccine development has been restricted to a relatively small number of parasite proteins, the most successful of which has an efficacy of 30 - 40% (49). Although many novel antigens have been identified as potential targets of protective immunity, the majority have yet to be studied in the requisite depth required for enhanced vaccine design. Furthermore, despite the fact that antigenic diversity significantly hampers the effectiveness of many vaccines including malaria, comprehensive approaches to determine the variants that could induce broad protection are challenging and consequently rarely undertaken. Here we focus on the relatively understudied malaria vaccine candidate ARMA. We present a systematic strategy that combines contemporary sequence data and measures of functional immunity with corresponding population level sero-epidemiology in diverse geographical locations to deduce the handful of antigen variants with the greatest potential to induce broad protective immunity. Our stepwise analytical approach could be applied to the wide range of complex pathogens for which antigenic diversity limits the design of highly effective vaccines.

Rigorous analyses of the *arma* locus in a sizable selection (n = 2,317) of nucleotide sequences of *P. falciparum* isolates from 13 countries revealed marked population structuring between Africa and Asia, consistent with previous reports (50–53). In contrast, the African haplotypes were strongly related and the level of nucleotide diversity did not vary by country as reported previously for other antigen genes (50). Importantly, although we found a dauntingly high number of haplotypes in Africa, the majority occurred at low frequencies (singletons, ~73%), and masked the only two that were actually dominant. Remarkably, this pair of haplotypes differed at a single polymorphic site (position 68) in the N-terminal region that appeared to be under balancing selection despite a negative Tajima’s D value (indicating directional selection) for the entire gene. These findings suggested that recombinant proteins based on just two haplotypes may be sufficient to induce broad protection against malaria. We also identified a highly conserved region (80 aa) contiguous to the polymorphic N-terminus that could provide additional opportunities for the design of a vaccine that would not be limited by antigenic diversity. However, this latter strategy that focuses on conserved epitopes seems to be less immunogenic and proved unsuccessful for the well-studied malaria antigen MSP1 (54).

We demonstrate for the first time the colocalization of ARMA with MSP1, a GPI-anchored protein containing a cysteine-rich domain. Our findings support previous *in silico* predictions (55), and reveal a C-terminal GPI modification site similar to that found in other merozoite surface proteins (23, 56). The detection of multiple bands in western blots indicates proteolytic processing (21, 56–58) and could result in oligomers of ARMA or complexes with other surface proteins (23, 59). Surface localization favors the hypothesis that ARMA is accessible to antibodies, and we demonstrated the induction of Fc-dependent (60) and independent mechanisms of parasite inhibition. We observed a degree of strain-specific functional antibody activity against the two dominant strains that differ at just a single amino acid site. Thus, given that the majority of epitopes are conserved, the inclusion of both dominant haplotypes in a vaccine construct can be predicted to induce functional antibodies against most parasite strains. The Fc-mediated activities of antibodies occurred selectively through antibody-dependent cellular cytotoxicity by NK cells in a dose-dependent manner in our study. The level of NK cell degranulation in variant V1 was comparable to that of MSP1. This activity has been recently demonstrated to be an important mechanism of action of antibodies in malaria in the context of both naturally acquired immunity and vaccine studies (35, 60, 61)

We detected extensive variation in the serological responses to the 27 common variants of ARMA identified in African isolates. This level of diversity was remarkable given that there were only 1 - 4 amino acid differences between variants. However, a hierarchical clustering analysis revealed only three serogroups that were similar for both IgG and IgM antibodies. Although the serogroups (C1-C3) were evenly distributed across the three African countries analyzed, population response profiles reflected geographical location. Serogroup C3 comprised two-thirds of the variants (18/27) and included both dominant variants (V1 and V2), and comparable representation of C204/G204 haplotypes (9:11). Antibody responses within serogroup 3 were highly correlated, providing further evidence that the selection of variants within this serogroup could lead to broad protection.

Previous studies have focused mainly on antibodies of the IgG isotype, and investigated combinations of IgG antibodies against multiple but distinct antigens (9, 16, 22, 62, 63). Here, the combination of IgG and IgM to different variants of the same antigen was the strongest predictor of protection in all three geographical locations. Our data suggest that despite the extensive serological diversity we observed in this study, only a handful of variants may be sufficient to induce effective immunity when both isotypes are considered. Thus, an analysis incorporating both isotypes appears to be more instructive than those that focus on either isotype alone (55, 64–67).

Mechanistically, antibodies of both isotypes may bind to different parts of the same antigen (67), maximizing complementarity in function. IgM has been demonstrated to be more effective at complement activation (68) and binds a different F_C_ receptor (69), while IgG is associated with increased affinity due to somatic hypermutation. Substantial functional studies, epitope mapping and additional analysis in animal models are needed to further understand how these interactions promote parasite clearance. Antibodies of the IgM isotype are typically produced first in response to infections (70–72), and replaced by IgG after affinity maturation and isotype switching during secondary exposure. Our findings reinforce recent evidence indicating that IgM antibodies have a role in malarial immunity (66, 73).

We found a strong correlation between IgM and IgG responses. Since IgM is pentameric, we anticipated that we would detect higher levels of this isotype compared to IgG. Our observation suggests that IgM was not fully “replaced” by IgG although individuals were exposed to malaria for a couple of years prior to sample collection. In accordance with this, the kinetics of IgM and IgG production was shown to be comparable in a controlled human malaria infection study and under conditions of natural exposure to malaria (66). Our data support the suggestion that the IgM response in malaria is not restricted to first exposures (66), and is likely due to the formation of memory IgM^+^ B cells (73). The maintenance of IgM production in response to malaria might suggest that IgG is not sufficient for effective parasite control. IgM antibodies against ARMA were associated with protection and mirrored those against IgG (6, 8, 74). Similarly, IgM responses to the whole merozoite and few other antigens, such as MSP1, MSP3 and AMA1 have been associated with protection from malaria in other studies (64, 66). Although we did not assess whether the IgM antibodies detected in our study were functional, anti-merozoite IgM has been shown to induce complement activity (66).

One limitation of our study is that sequence data was analyzed from the MalariaGEN Pf3k dataset and these geographic locations did not fully match the locations of our cohort studies. Notably, the dataset lacks East African isolates and may have resulted in our inability to identify suitable variants from Kisumu in Kenya. Genetic relatedness between parasites in West Africa has been reported previously (75, 76), and differs from that in East Africa (52, 75). Taken together, our findings suggest that the design of “universal” vaccine based on ARMA is best informed by a thorough analysis of the extent of the serological diversity in geographically distinct regions. Against this background, our data suggests that it will be possible to select a handful of variants that induce broad protection, such as that observed against V10, V22, V23 and V6 in our study.

Historically, the actual diversity of circulating parasite strains has not been factored into malaria vaccine design and may have contributed to consistently low efficacy (50). It is therefore prudent to analyze the impact of antigen diversity on cross-strain protective immunity before advancing to preclinical and clinical trials. Our systematic approach to the analysis of all the common variants of ARMA found in West and Central Africa, and their impact on serological diversity in multiple cohorts could serve as a model for other studies. The simultaneous analysis of combinations of IgM and IgG isotypes with regard to protection is novel, and offers new avenues to investigate protective immunity. Our strategy lends support to the idea that despite extensive variation and diversity, a small number of proteins can induce broad cross-strain protective immunity. Given the extensive flexibility of protein microarrays; our method can be further enhanced by the inclusion of multiple distinct antigens, additional variants and the systematic analysis of additional isotypes, including IgG subclasses.

In conclusion, our study suggests that both IgG and IgM antibodies against the merozoite surface antigen ARMA contribute to protection against malaria. When both isotypes are considered, only a handful of antigen variants appear to be sufficient to overcome the extensive genetic diversity observed in African isolates and bodes well for vaccine development. We provide an adaptable pipeline for the systematic analysis of antigen diversity in sero-epidemiological studies at the earliest stages of vaccine development.

## Data availability statement

The raw data supporting the conclusions of this article will be made available by the authors, without undue reservation.

## Ethics statement

The studies involving human participants were reviewed and approved by Kenya Medical Research Institute Scientific and Ethics Review Unit (KEMRI/RES/7/3/1). Written informed consent to participate in this study was provided by the participants’ legal guardian/next of kin.

## Author contributions

FO, JR and KN conceived the study, performed formal analysis and interpreted the data. KN and JT designed the DNA constructs. KN cloned the expression vectors. KN, TC and JT performed protein expression and microarray design, printing and processing. KM. cleaned and normalized the data. KN and KM performed the statistical analysis of the data. KN performed the computational analysis. MR, ZS, DO and KN performed Immunological experiments and immunofluorescence assays. LN aided in coordinating shipment and procurement. KN drafted the original manuscript. KN, JR and FO did major review and editing of the manuscript. AT, JW, BO, SS and BK contributed with samples and clinical data. GA, BK, JR and FO. supervised. GA and FO obtained funding. FO coordinated projects. All authors contributed to the article and approved the submitted version.
