# Supplementary material for: Characterization of a novel Plasmodium falciparum merozoite surface antigen and potential vaccine target
Source: Front Immunol. 2023 Apr 14;14:1156806. doi: 10.3389/fimmu.2023.1156806 (PMC10140549; doi:10.3389/fimmu.2023.1156806)
Supplement: Supplementary file 1 [file DataSheet_1.pdf]

Supplementary Materials for

Characterization of a novel *Plasmodium falciparum* merozoite surface antigen and potential vaccine target

Karamoko Niaré et al.

Corresponding authors: karaniare@icermali.org (KN); fosier@kemri-wellcome.org (F.H.A.O)

MATERIALS AND METHODS

Merozoite enzyme-linked immunosorbent assay

To extract merozoites, infected red blood cells were monitored until most of the parasites reached the early schizont stage. Cysteine-protease inhibitor E46 was added to a final concentration of 10 µM to prevent cell rupture during schizont maturation. After 6 – 8 hours, merozoites were isolated by pushing the culture through a 1.2 µm syringe filter. After centrifugation, the filtrate was resuspended in PBS and the merozoite density was measured by flow cytometry. The suspension was further diluted in PBS supplemented with a protease inhibitor to reach a working concentration of 15 x 10^6 merozoites/mL. We used a 2-day ELISA protocol to test the specificity of our rabbit antisera. Briefly, the merozoite suspension was coated in a 96-well flat-bottom plate (100 µL/well) for an overnight incubation at 4 °C. The wash steps were performed with PBS. For blocking, 0.1 % casein in PBS was added (200 µL/well) and the plate was incubated for 2 hours at 37 °C. A serial dilution of the rabbit antisera ranging from

12 to 7.3 x 10^{-4} mg/ml was added to the plate (50 µL/well) in duplicates for 2-hour incubation at 37 ° C. After wash, the plate was incubated with diluted goat HRP-conjugated anti-rabbit IgG (1:2500, 50 µL/well) for 1 hour at 37 °C. The rest of the experiment was performed as described above in antigen ELISA (Materials and Methods section).

Rabbit immunization

We generated recombinant proteins (V1, V2 and CD4) at Kenya Medical Research Institute (KEMRI) – Wellcome Trust Research Programme in Kilifi, Kenya and sent them to BioGenes GmbH company based in Berlin, Germany, to raise antibodies in rabbits using a custom 35-day immunization protocol. Two Zimmermann rabbits were immunized per protein. Rabbits were first bled on day 0 to obtain pre-immune sera before the first injection with 0.5 mg of protein. On day 21, they received a boost with 0.25 mg of protein and were kept for two more weeks. The final bleeding was performed on day 35 and the total IgG antibodies were isolated using protein A column.

C1q fixation assay

This assay was performed using a one-day ELISA protocol. Four concentrations (30, 10, 3 and 1 µg/mL) of the antigens (V1, V2, AMA1 and CD4) were coated in 96-well plates (100 µL/well) for 2 hours at 37°C. Plates were washed with PBST before blocking with 10% skimmed milk in PBST for 2 hours. Diluted MIG, PHIS, and German naïve serum (1:40) were added to the plates (50 µL/well) and incubated for 2 hours at room temperature. After washing, plates were incubated with 10 µg/mL of purified human C1q (Abcam) in 5% skimmed milk in PBST (40 µL/well) for 30 minutes at room temperature. Plates were later washed before adding diluted

sheep HRP-conjugated anti-C1q antibodies (1:200) in 5% skimmed milk in PBST (50µL/well). Following an incubation at room temperature for 1 hour, the reactions were developed as described above in antigen ELISA.

Antibody-dependent respiratory burst assay

To isolate neutrophils, fresh blood (~ 60 mL) was mixed with Hanks buffered salt solution (HBSS, 1:1) and gently layered on top of Histopaque-1077 (Sigma-Aldrich) before centrifugation at 600 x g for 15 minutes. The peripheral blood mononuclear cells and plasma were removed and the cell pellets were resuspended in 5 mL of HBSS. The suspension was mixed with 3 % dextran in PBS (1:2) and incubated at room temperature for 1 hour. Thereafter, the supernatant was collected after decantation and centrifuged at 500 x g for 7 min at 4 °C. The supernatant was discarded and the residual red blood cells in the pellet were lysed by adding cold 0.2 % NaCl for 30 seconds followed by neutralization with an equal volume of cold 1.6 % NaCl. The neutrophils that remained were resuspended in sterile 0.1 % BSA, 1 % D-glucose in HBSS to obtain to 1 x 10^{17} /mL.

The ADRB assay was performed as previously described (Llewellyn et al., 2015). Briefly, the same concentrations of antigens (V1, V2, AMA1 and CD4) were coated in Nunc opaque Maxisorp 96-well plates (Thermo Scientific) as described above in antigen ELISA. Plates were washed with sterile PBS and blocked with 1 % casein in BST (200 µL/well) for 1 hour at room temperature. After washing the plates, diluted MIG, PHIS and naïve serum in PBST (1:40) were added (50 µL/well) before incubating at 37 °C for 1 hour. Finally, 0.04 mg/mL luminol (50

µL/well) and 1 x 10^7 isolated neutrophils (50 µL/well) were quickly added after wash and the chemiluminescence was immediately measured using Synergy 4 plate reader (Biotek).

Quality control of protein microarray data

Although the normalization helps to correct for systematic variations in protein microarrays, producing quality raw data is key. To minimize technical and systematic errors, we took different quality control actions during the microarray design and processing including: i) the use of various types of control antibodies and sera, ii) the reduction of the number of sample batches, iii) random selection of samples from different sites to be used onto the same chips and, iv) limitation of the number of persons involved in sample processing and data extraction.

The median fluorescence intensity (MFI) values of the buffer spots were very low (median ≈ 0) with minimal chip-to-chip and day-to-day variations (Supplementary Figure 9), suggesting low non-specific antibody binding and quality sample processing. The background signals were generally low despite some outliers (Supplementary Figure 10) corresponding to 41 samples (5.7%) that looked degraded on the mini-array scans (Supplementary Figure 11). There was no significant difference in the background signal variations between antigens (Supplementary Figure 10). The buffer signals were low and consistent across days of sample processing (Supplementary Figure 12). After removing these outliers, we remained with 675 samples for all the downstream analyses. We observed negligible intra-assay variations and MFI values from replicate spots were strongly correlated (P-value <0.001, R=0.9927, 95% CI [0.9926-0.9929] for IgG; and P-value < 0.001, R=0.9877, 95% CI [0.9875-0.9880] for IgM) (Supplementary Figure 13)

for both antigens and controls. A principal component analysis of the raw data of all the antigens showed clear separation of the naive controls and blanks from PHIS (Supplementary Figure 14). As expected, the test samples that included seronegative subjects, low and high responders spread out into both PHIS and naive control clusters. Altogether, the data structure denoted minimal technical variations in general. Only variations in antibody reactivities among high responders were systematically higher compared to low responders (Supplementary Figure 15). To deal with these common systematic variations in protein microarrays and batch effects while preserving biological differences between samples before statistical analyses, we normalized the data using combined ComBat and variance stabilizing normalization (vsn) methods (*1*–*3*). These normalization approaches could correct effectively for the systematic high variations in high responders (Supplementary Figure 16). Overall, the quality of the IgG and IgM responses was highly comparable.

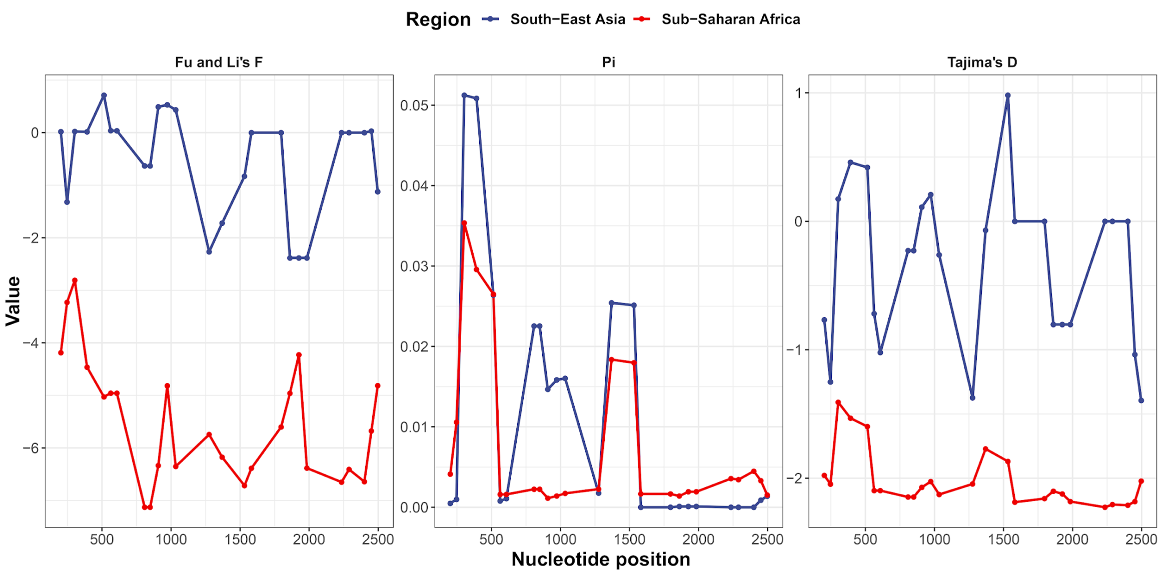

Supplementary Figure 1. Selection pressure along the *Pf3D7_1136200* gene in West and Central Africa (*N = 1,333*) and South-East Asia (n = 984). Pi is the nucleotide diversity (π). Tajima's D and Fu and Li's F are neutrality tests.

A

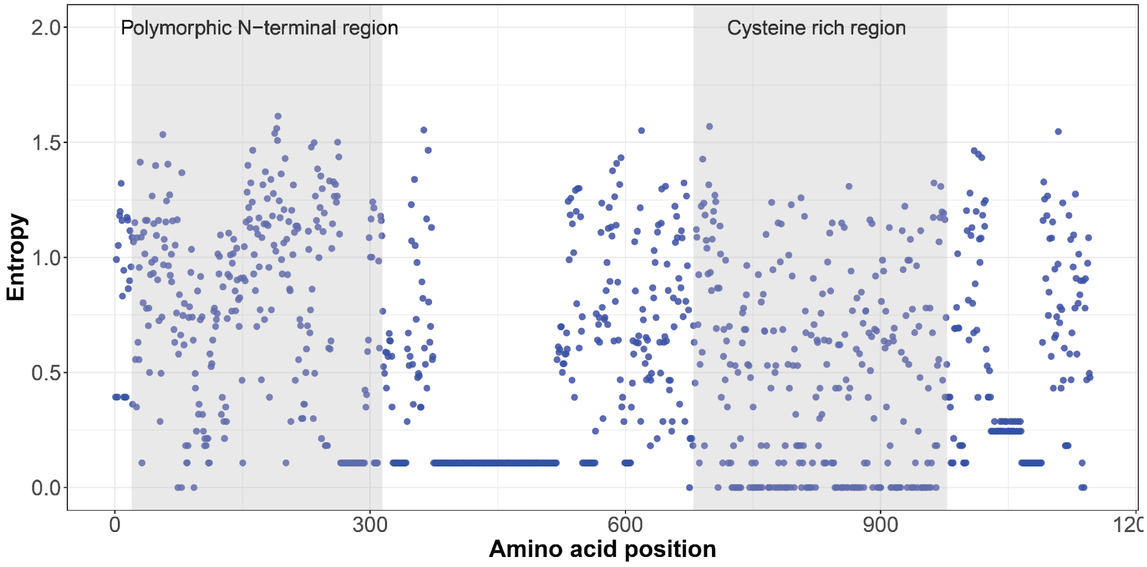

B

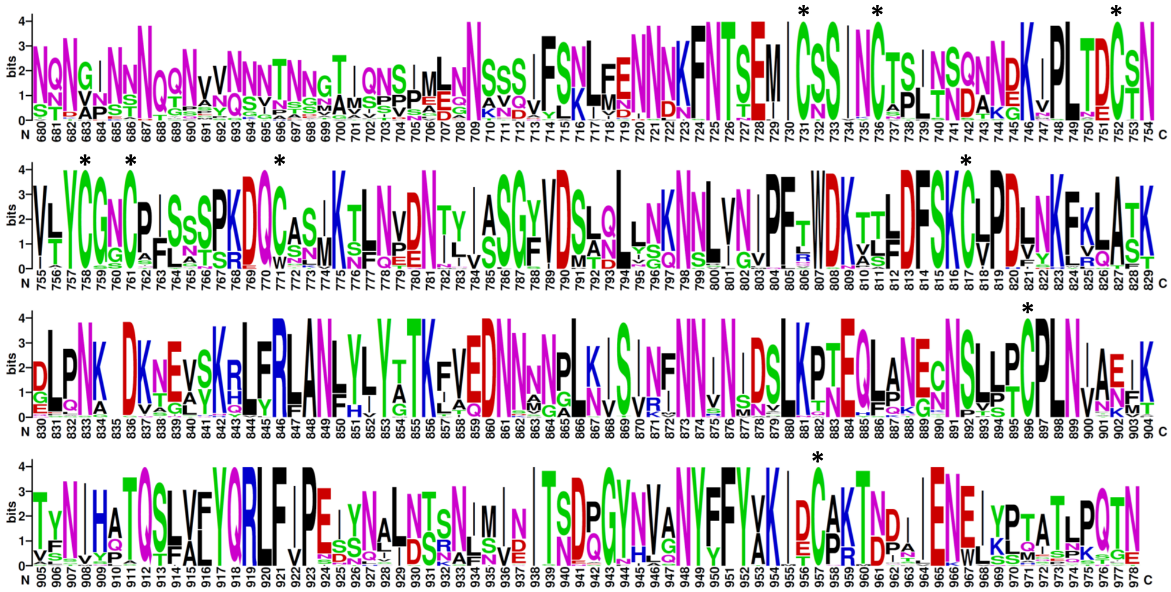

Supplementary Figure 2. Conservation analysis of PF3D7_1136200 across different *Plasmodium* species. This analysis comprised 45 orthologous sequences from 17 different *Plasmodium* species that were aligned to each other. **A)** Shannon entropies of all amino acid positions**.** Each dot represents a unique amino acid position. Entropy value of 0 at an amino acid position refers to 100% conservation across all species. The mean entropy value for the C-terminal cysteine rich region was 0.43. **B)** Sequence logo of the C-terminal region. This analysis showed a high level of amino acid conservation. The 9 conserved cysteine residues are marked by asterisks.

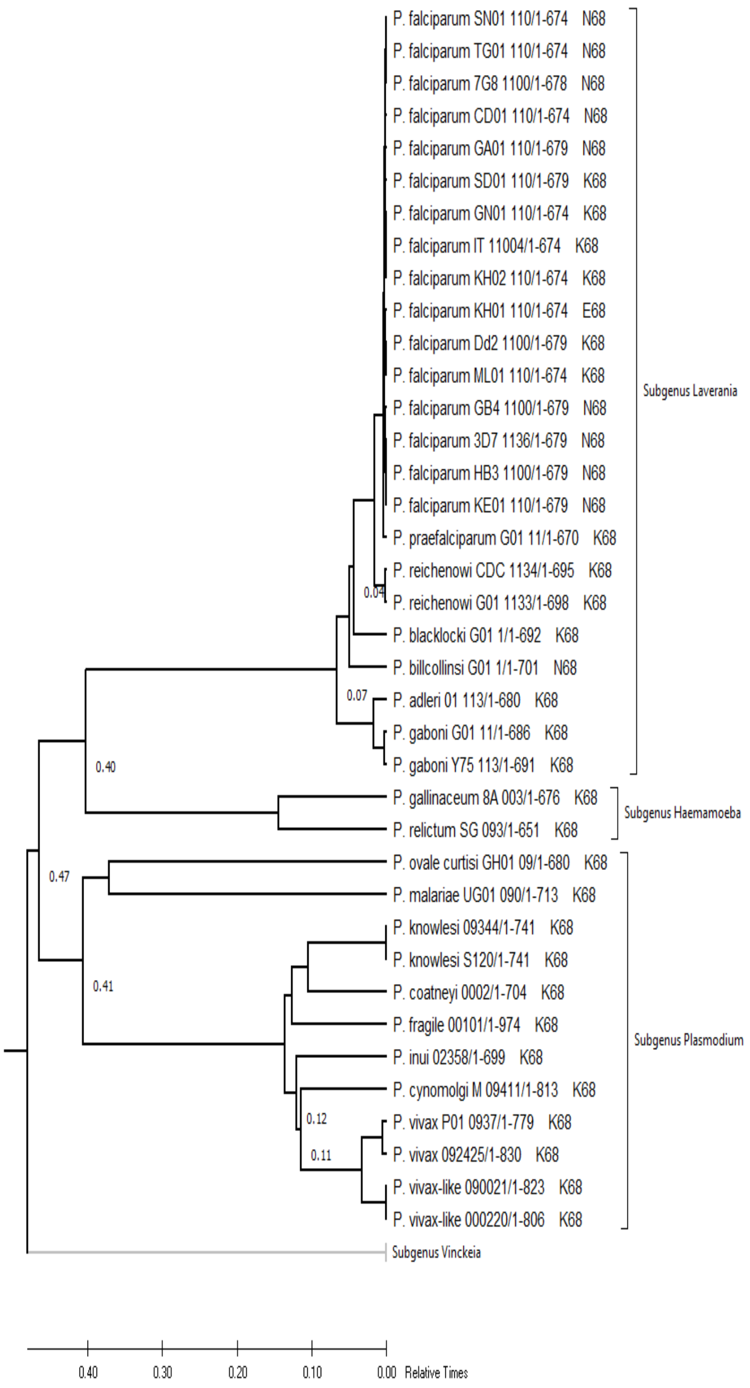

Supplementary Figure 34. Ancestral relationship between orthologous sequences. Data (45 PF3D7_1136200 sequences from 16 *P. falciparum* strains and 20 other *Plasmodium* species) was

analyzed by the unweighted pair group method with arithmetic mean. The outcomes of the timetree analysis are displayed on tree branches. The nature of the allele at position 68 (N68 or K68) is added to sequence labels. The allele N68 occurs only in members of *Laverania* species. The *Vinckeia* sub-genus was defined as an outgroup.

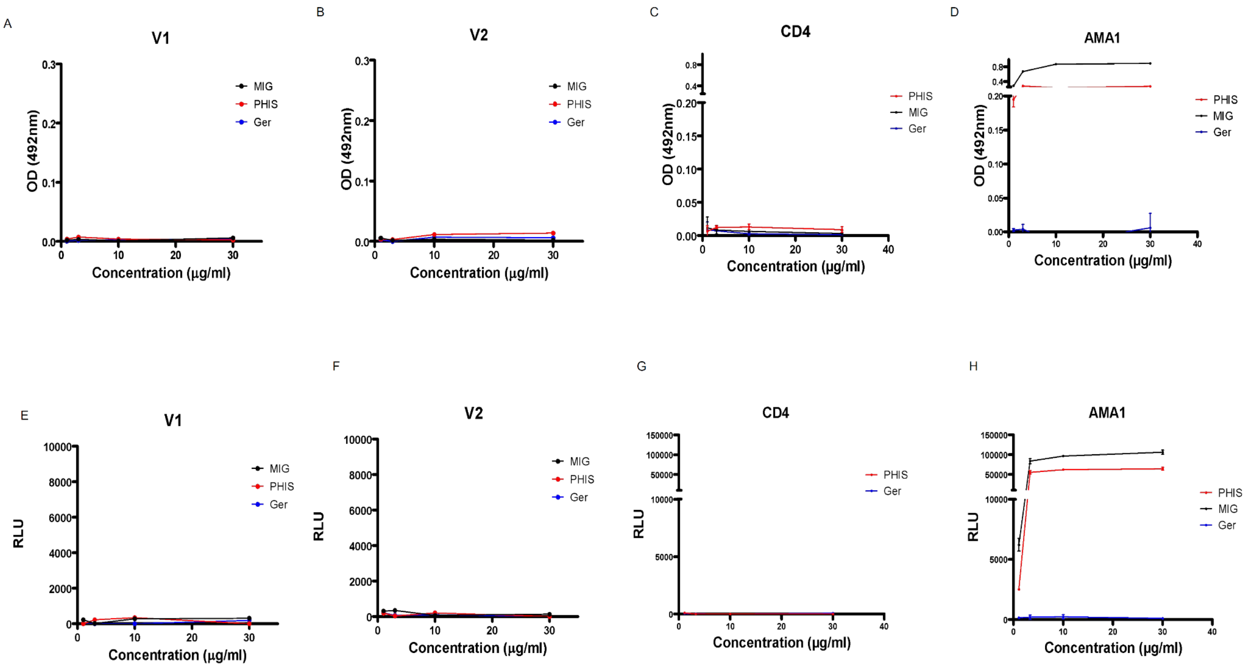

Supplementary Figure 4. Outcomes of Fc-dependent antibody function assays against PF3D7_1136200 variants. **A)**, **B)**, **C)** and **D)** C1q fixation assay using V1, V2, CD4 and AMA1; respectively. Only antibodies targeting AMA1 in PHIS and MIG fixed C1q in dose-dependent manner. **E)**, **F)**, **G)** and **H)** Antibody-dependent respiratory burst assay using V1, V2, CD4 and AMA1; respectively. Only anti-AMA1 antibodies in PHIS and MIG showed activity which was dose-dependent

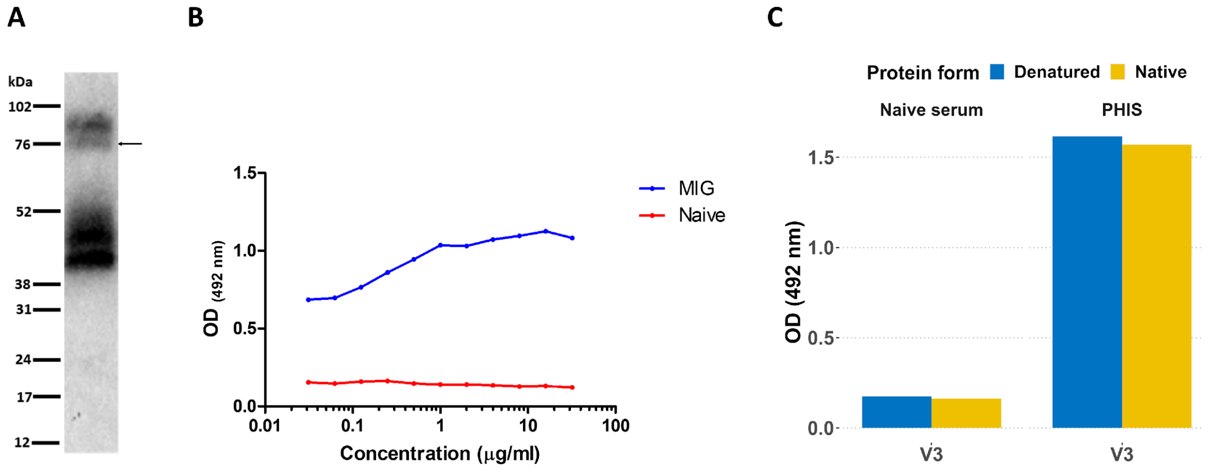

Supplementary Figure 5. Quality of the recombinant proteins. **(A)** Western blot of an illustrative variant of PF3D7_1136200, V3. Experiments were performed with 10 µl of purified protein. Proteins were fragmented after expression. The size of the full-length protein was ~ 75 kDa (indicated by an arrow) **(B)** ELISA standard curve. MIG: purified malaria immunoglobulins. Naive: malaria-naive serum. **(C)** ELISA of the native and denatured forms of V3. PHIS: pooled malaria hyperimmune sera. Proteins were heat-denatured at 70°C for 1 hour and coated at 1 µg/ml.

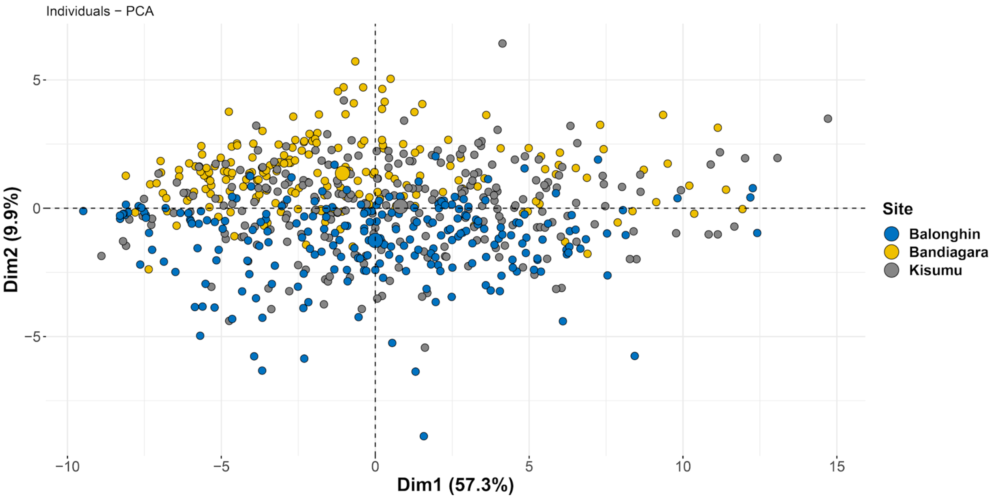

Supplementary Figure 6. Principal component analysis of normalized IgG reactivity data. Data includes MFI values of samples from all three sites (*N = 675*). IgM showed similar patterns.

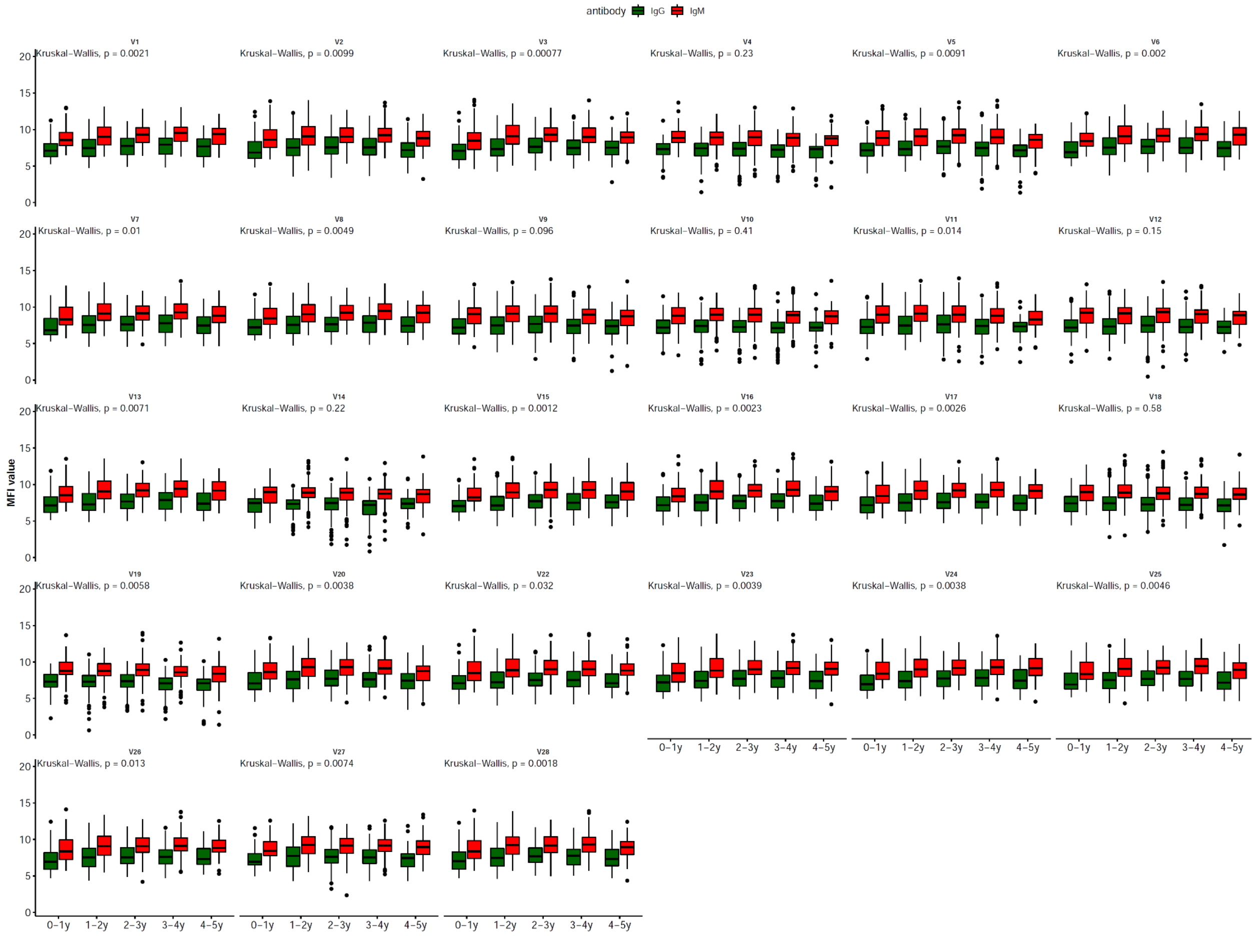

Supplementary Figure 7: Antibody reactivities and age. Children were categorized into five age groups: 0 – 1 years, 1 –7 2 years, 2 – 3 years, 3 – 4 years and 4 – 5 years. P-values are for IgG and IgM analyzed together. When analyzed separately, magnitudes of IgG against 7 of 27 variants and IgM against 14 of 27 variants increased significantly with age. IgG and IgM responses to V19 and V20 decreased significantly with age, respectively.

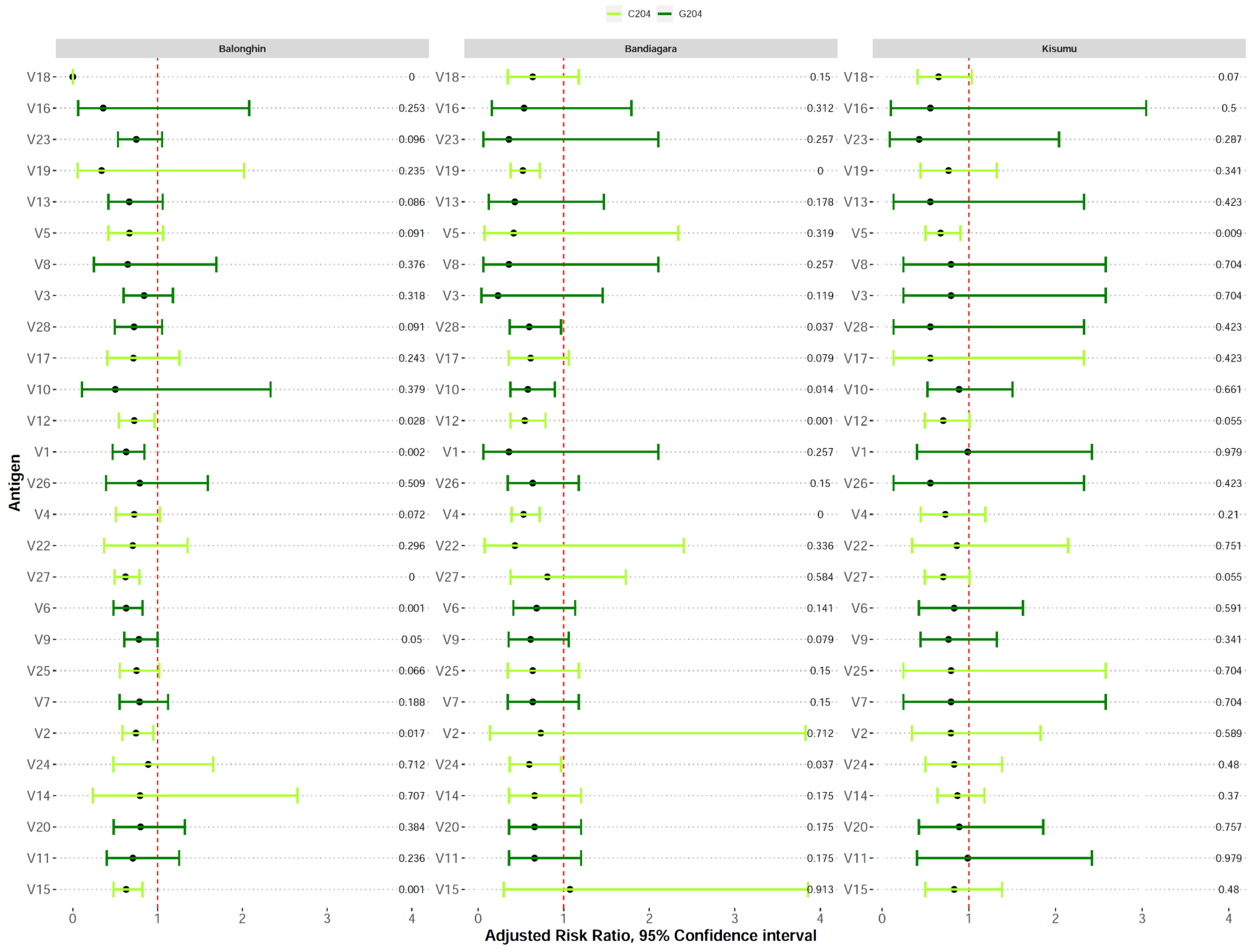

Supplementary Figure 8. Associations between IgM against PF3D7_1136200 variants and protection from malaria. Risk ratios showing strengths of associations between single IgM responses and protection from clinical episodes of malaria in all three sites

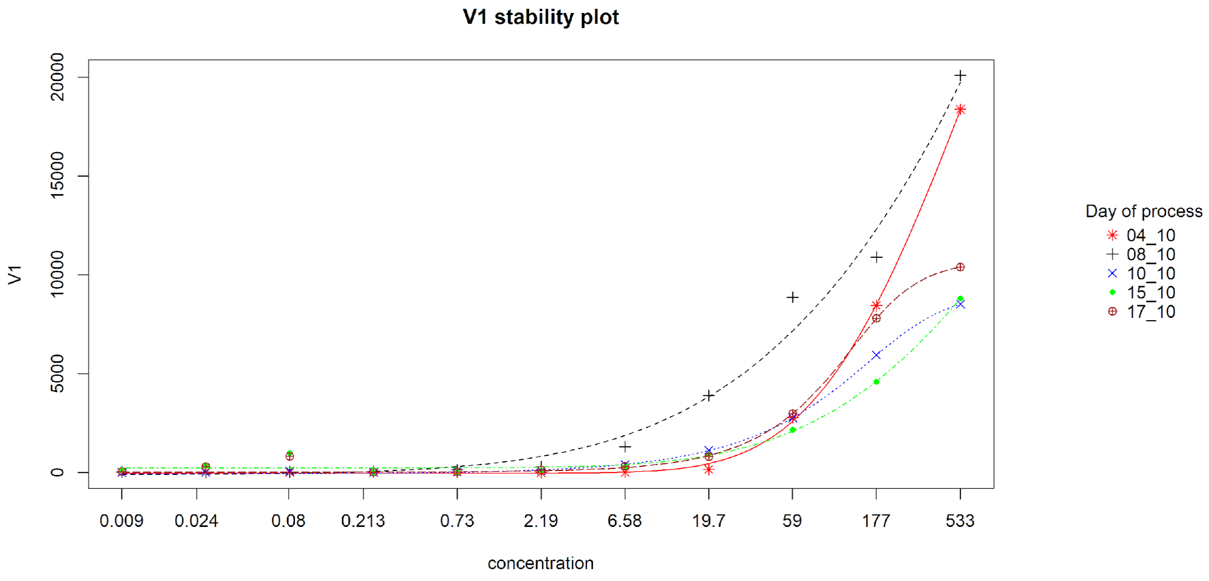

Supplementary Figure 9. Day-to-day variation using purified malaria immunoglobulin (MIG) dilutions against an illustrative antigen variant (V1). Concentrations ranged between 0.009 and 533 µg/mL (x-axis). Mean fluorescence intensity (MFI) of V1 represents the y-axis. The titration was performed during every run of microarray processing.

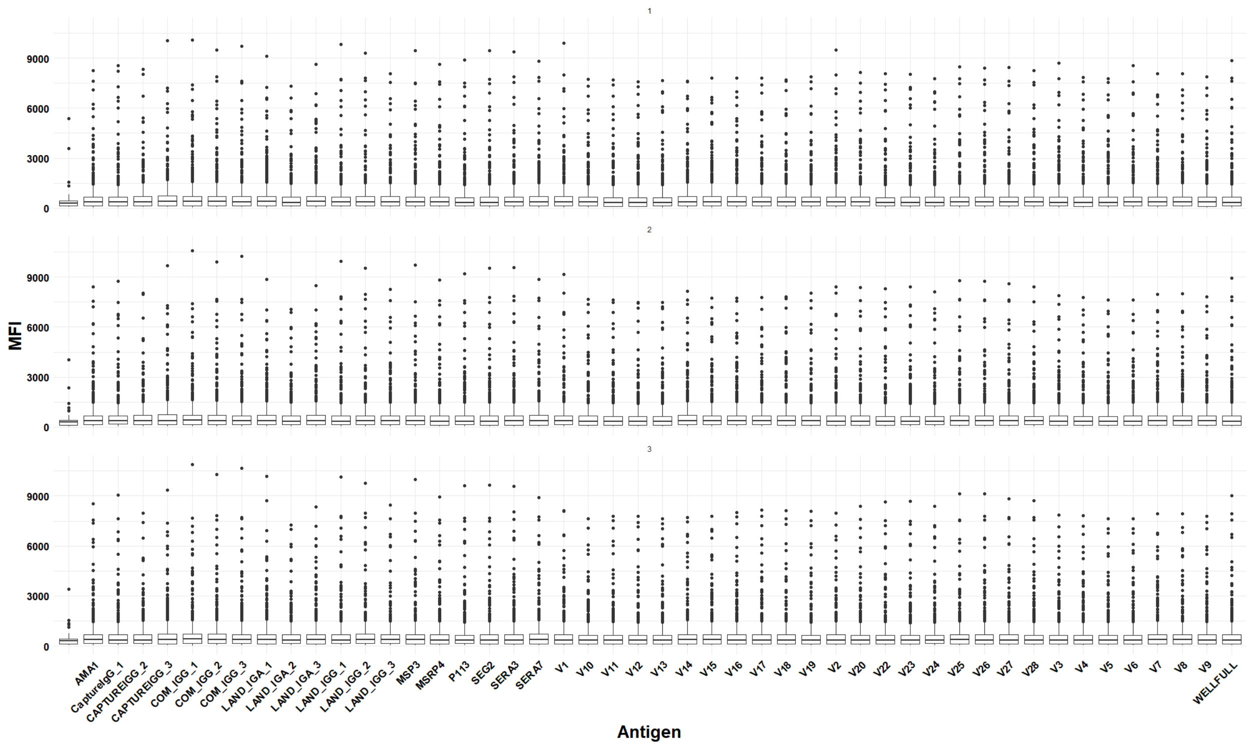

Supplementary Figure 10. Distribution of microarray background signals. The median background MFI values were very low and consistent across antigens and control antibodies that were printed onto the chip. The outliers represent the degraded slide images which were removed before prior to data analysis.

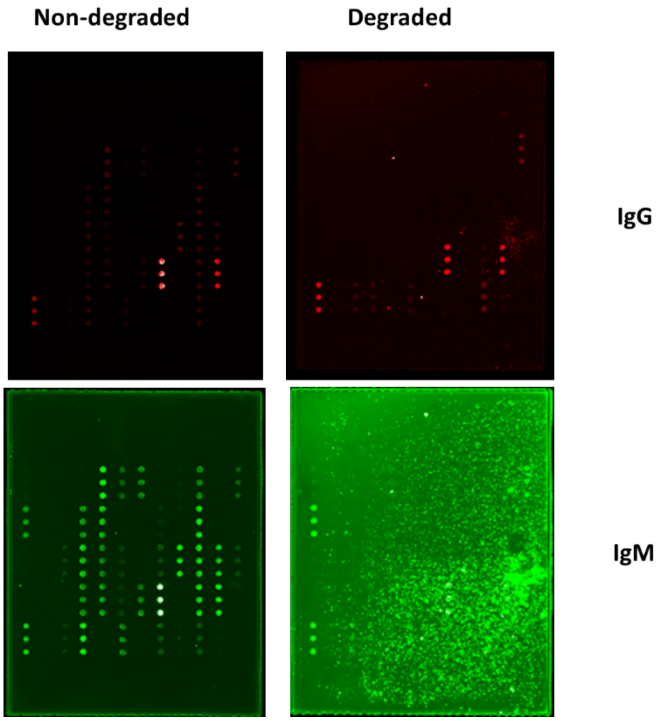

Supplementary Figure 11. Quality of illustrative miniarray images scanned after sample processing. Red and green images correspond to the same miniarrays scanned at 635 nm (IgG) and 532 nm (IgM), respectively.

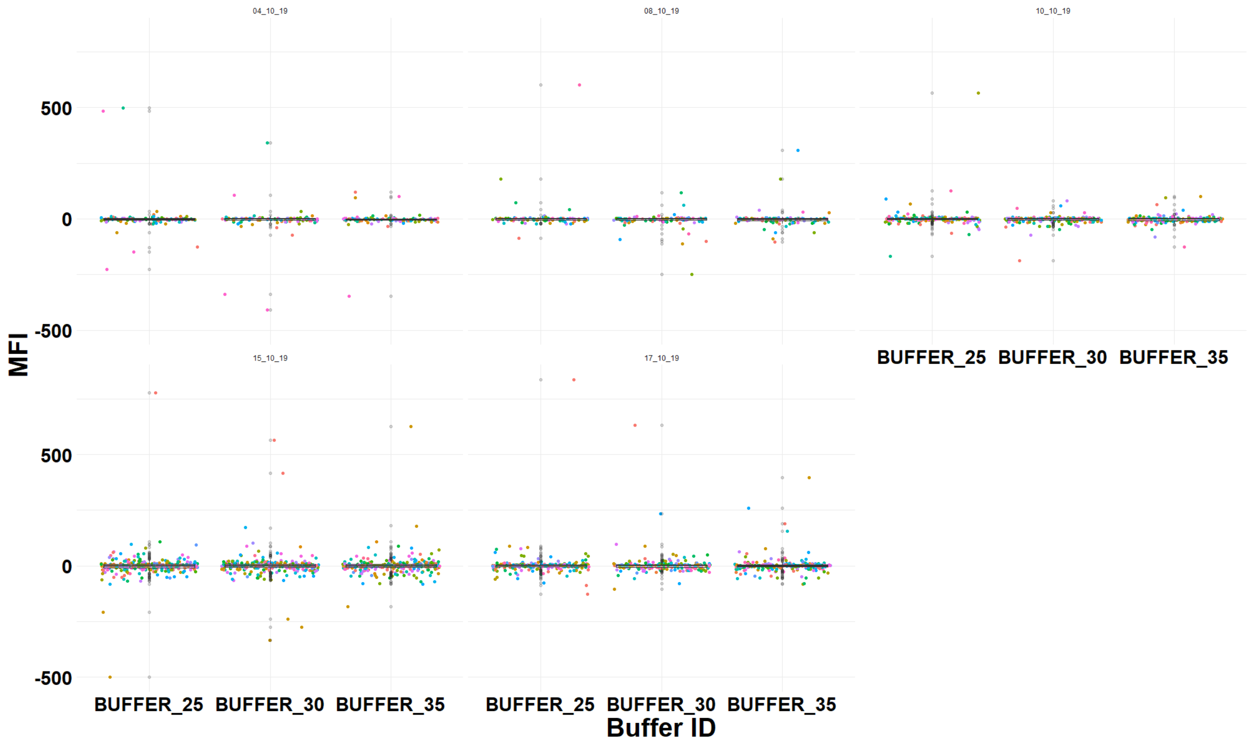

Supplementary Figure 12. Distribution of buffer spot mean fluorescence intensities by day of sample processing. The buffer signals were very low (close to zero) and consistent by day. Three sets of buffer spots in triplicates (buffer spot set IDs: 25, 30 and 35) were used in each run of sample processing.

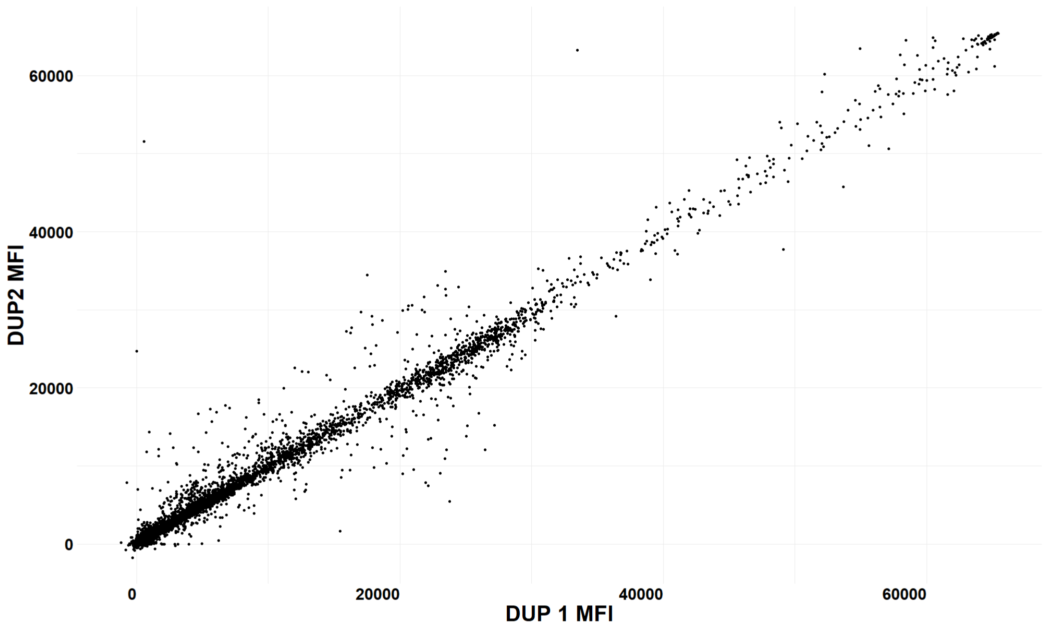

Supplementary Figure 13. Replicate analysis. MFI values of the first two spots (duplicate 1 [DUP1] and 2 [DUP2]) are compared. This plot represents IgG data which were highly comparable to those of IgM. The two spots were highly correlated (for IgG: R = 0.9927 [0.9926 – 0.9929], $P < 0.001$; for IgM: R = 0.9877 [0.9875 – 0.9880], $P < 0.001$).

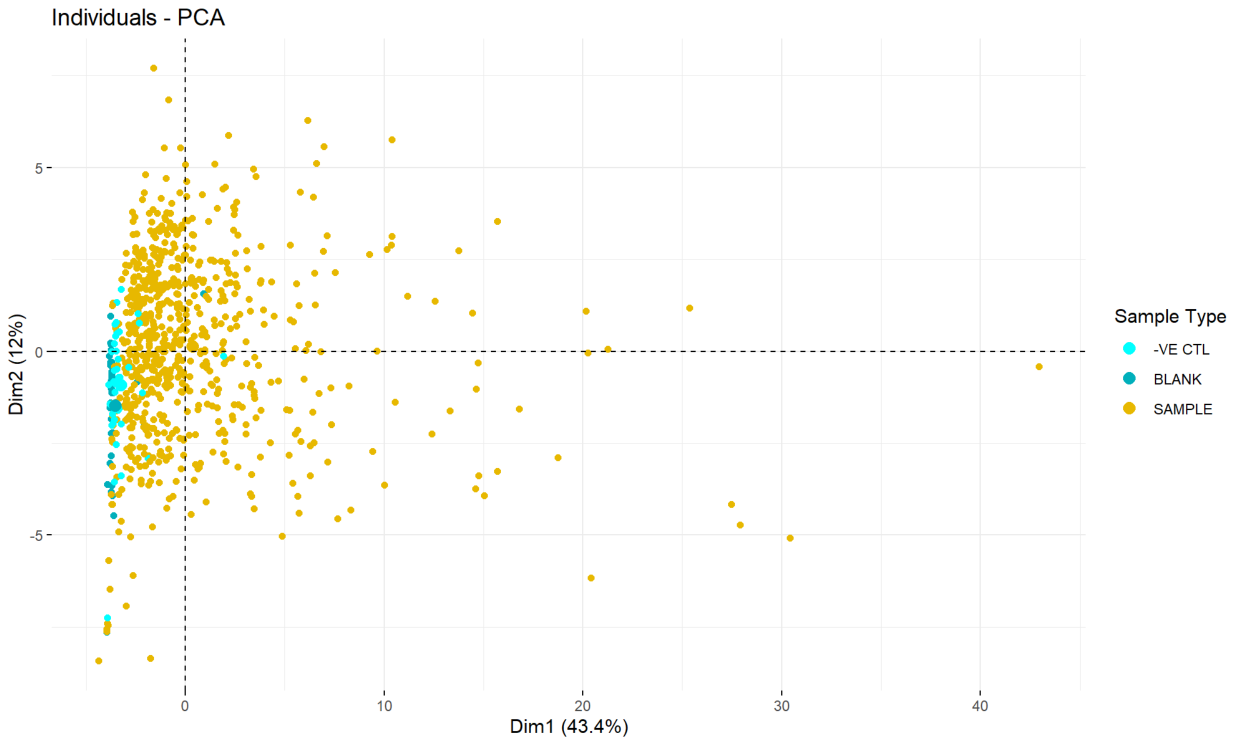

Supplementary Figure 14: Principal component analysis of the raw antibody reactivity data showing the difference between samples and negative controls. Blank represents the spotted printing buffers. -VE CTL (negative controls) represents 22 malaria-naive sera from Europe.

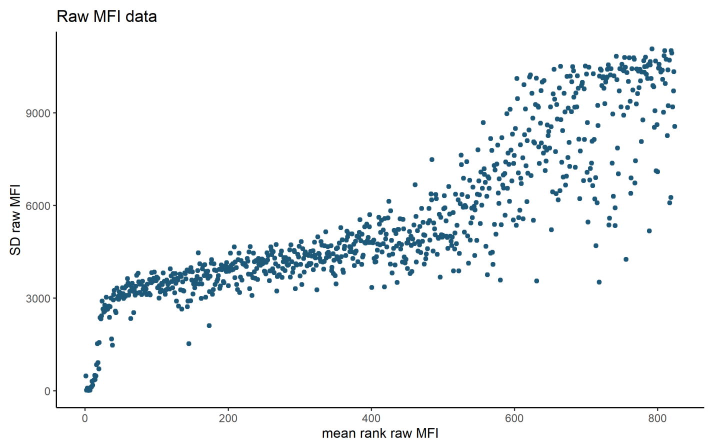

Supplementary Figure 15: Distribution of inter-individual MFI variations before data normalization. The standard deviations (SDs) were high in high responders.

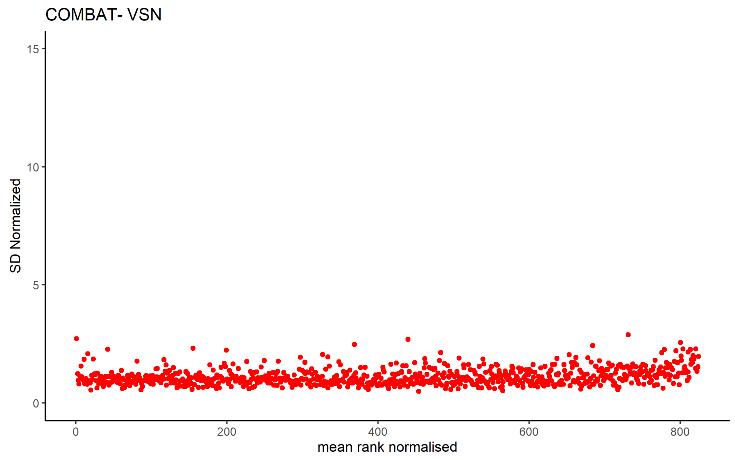

Fig. 16: Distribution of inter-individual MFI variations after data normalization. The high standard deviations (SDs) observed in high responders were drastically reduced by the double normalization approaches. COMBAT: a normalization approach that adjusts for batch effects using empirical Bayes framework. VSN: Variance stabilizing normalization.

Supplementary Table 1: Estimates of genetic diversity and selection pressure in West and Central Africa.

Country	Eta	Hap	Hd	π (10^{-3})	Tajima's D	*P*	Fu & Li's F	*P*	Samples analyzed (*n*)
DRC	18	20	0.69	4.76	-2.02	<0.05	-3.63	< 0.02	106
The Gambia	9	10	0.69	4.53	-1.41	> 0.10	-2.71	< 0.05	58
Ghana	57	63	0.70	4.82	-2.44	< 0.001	-8.14	<0.02	569
Guinea	16	18	0.65	4.33	-1.98	< 0.05	-3.95	<0.02	100
Malawi	32	39	0.67	4.56	-2.19	< 0.01	-4.30	< 0.02	363
Mali	13	13	0.68	4.54	-1.86	< 0.05	-3.85	< 0.02	68
Senegal	14	13	0.69	4.79	-1.90	< 0.05	-3.64	< 0.02	69
Overall	111	125	0.69	4.69	-2.57	ND	-9.69	< 0.02	1333

Tajima's D and Fu and Li's F values were significantly correlated (Pearson's R = 0.73, p = 0.025). DRC: Democratic Republic of Congo. Eta: total number of mutations (nonsynonymous only). Hap: number of haplotypes. Hd: haplotype diversity. π: nucleotide diversity.

Supplementary Table 2: List of PF3D7_1136200 variants printed onto protein microarray.

Variant name	Amino acid position	Reference amino acid	Mutated amino acid	Haplotype family	Frequency (%)
V1	68	N	K	G204	38.9
V2 (3D7)*	N/A	N/A	N/A	C204	40.3
V3	60,68	V,N	A,K	G204	2.4
V4	60	V	A	C204	0.5
V5	75	F	I	C204	0.7
V6	68,191	N,I	K,K	G204	0.3
V7	68,575	N,F	K,I	G204	0.5
V8	63,68	T,N	M,K	G204	2.6
V9	61,68	P,N	S,K	G204	0.2
V10	67,68	N,N	K,K	G204	0.3
V11	61,68	P,N	L,K	G204	0.9
V12	648	T	I	C204	0.2
V13	68,116	N,R	K,K	G204	0.5
V14	339	I	T	C204	0.2
V15	63	T	M	C204	0.2
V16	68,339	N,I	K,T	G204	0.2
V17	631	N	H	C204	0.2
V18	116	P	S	C204	0.2
V19	191	I	K	C204	0.5
V20	68,510	N,Q	K,P	G204	0.2
V22	61	P	L,K	C204	0.2
V23	58,68	D,N	H,K	G204	0.2
V24	526	A	G	C204	0.2
V25	486	N	K	C204	0.2
V26	63,68	T,N	A,K	G204	0.2
V27	33	D	Y	C204	0.2
V28	60,68,131	V,N,I	A,K,T	G204	0.2

*Reference sequence, same as the 3D7 version. Variants 21, 29, and 30 were excluded as they had mutations within the GPI modification site which was removed prior to protein expression. N/A: not application.

References

1. W. Huber, A. Von Heydebreck, H. Sültmann, A. Poustka, M. Vingron, Variance stabilization applied to microarray data calibration and to the quantification of differential expression. *Bioinformatics*. **18** (2002), doi:10.1093/bioinformatics/18.suppl_1.S96.

2. W. E. Johnson, C. Li, A. Rabinovic, Adjusting batch effects in microarray expression data using empirical Bayes methods. *Biostatistics*. **8**, 118–127 (2007).

3. G. Kamuyu, J. Tuju, R. Kimathi, K. Mwai, J. Mburu, N. Kibinge, M. Chong Kwan, S. Hawkings, R. Yaa, E. Chepsat, J. M. Njunge, T. Chege, F. Guleid, M. Rosenkranz, C. K. Kariuki, R. Frank, S. M. Kinyanjui, L. M. Murungi, P. Bejon, A. Färnert, K. K. A. Tetteh, J. G. Beeson, D. J. Conway, K. Marsh, J. C. Rayner, F. H. A. Osier, KILchip v1.0: A Novel Plasmodium falciparum Merozoite Protein Microarray to Facilitate Malaria Vaccine Candidate Prioritization. *Front. Immunol.* **9**, 2866 (2018).
